# Catalytic mechanism and transcriptional regulation of AtsTPS2 involved in germacrene A biosynthesis in *Acorus tatarinowii*

**DOI:** 10.3389/fpls.2026.1761957

**Published:** 2026-04-29

**Authors:** Fayuan Peng, Jin Chu, Xiaofen Bai, Bo Wang, Jie Li, Xu Sun, Lin Liu, Honghong Fan

**Affiliations:** 1 School of Life Sciences, Anhui Agricultural University, Hefei, China; 2 Jinzhai County Traditional Chinese Medicine Industry Development Center, Lu’an, China; 3 Integrated Experimental Station in Dabie Mountains, Anhui Agricultural University, Lu’an, China

**Keywords:** *Acorus tatarinowii*, AtsMYC5, germacrene A, insect repellency, terpene synthase

## Abstract

*Acorus tatarinowii* Schott is a traditional medicinal plant valued for its mosquito-repellent properties, which are attributed to its terpenoid constituents. HS-SPME-GC-MS analysis of different tissues and developmental stages revealed a complex volatile profile, with multiple terpenoids detected in the samples. Genome-wide characterization of the terpene synthase (TPS) family revealed that the TPS-a subfamily forms a gene cluster on chromosome 6, likely resulting from tandem duplication events followed by relaxed selection pressure in tandem repeat regions. Functional assays showed that only *AtsTPS2* and *AtsTPS8* exhibit sesquiterpene synthase activity. Under HS-SPME-GC-MS conditions, AtsTPS8 produced germacrene B (which is detected as *γ*-elemene), whereas AtsTPS2 produced germacrene A (which is detected as *β*-elemene), these detected signals were interpreted as products of heat-induced rearrangement during analysis. *AtsTPS2* was selected for further study. Site-directed mutagenesis identified Lys461 as a critical residue regulating catalytic efficiency; compared with the wild type, the K461A mutant increased the yield of the detected product by 145%. Transient overexpression of AtsTPS2 in *A. tatarinowii* led to a 2.02-fold increase in the abundance of the detected product peak, and increased mosquito repellency to 83%. Furthermore, the MYC family transcription factor AtsMYC5 was found to repress *AtsTPS2* expression by directly binding to its promoter. This study identifies AtsTPS2 as a sesquiterpene synthase in *A. tatarinowii*, clarifies its catalytic properties and AtsMYC5-mediated transcriptional regulation, and suggests that the corresponding sesquiterpene pathway contributes to mosquito repellency in this species.

## 1 Introduction

*Acorus tatarinowii* Schott, a traditional medicinal plant, distributed from southern China to northeast India and northern Thailand, thrived predominantly in streamside wetlands (Cheng et al., 2020). This perennial herb typically matures over three years and is renowned for its pronounced mosquito-repellent properties and distinctive aromatic profile (Blythe et al., 2016), making it highly valued in traditional medicine (Hu et al., 2009; Su et al., 2022). The presence of numerous terpenoids in *Acorus tatarinowii* has been detected in previous studies. For example, more than 160 compounds spanning diverse structural types, including terpenoids, have been identified in *A. tatarinowii* (Wang et al., 2023). Analysis of the essential oil of *Acori Tatarinowii* Rhizoma (ATEO) from different regions of China by GC-MS revealed distinct volatile profiles and led to the identification of 57 constituents, a significant proportion of which were terpenoids (Yan et al., 2020). Similarly, a comparative GC-MS study integrated with chemometrics also identified a substantial number of volatile terpenoids when differentiating between *Acori Tatarinowii* Rhizoma and *Acori Calami* Rhizoma (Zhang et al., 2015). However, the functions and biosynthetic mechanisms of these volatile terpenoids in *A. tatarinowii* remain largely unknown.

Terpenoids, representing the most structurally diverse class of plant secondary metabolites (Gershenzon and Dudareva, 2007; Fan et al., 2021), play pivotal roles in both essential physiological processes and environmental stress adaptations (Pichersky and Raguso, 2018; Wei et al., 2024). Terpenoids are mainly classified into monoterpenes, sesquiterpenes, diterpenes, and other categories. Based on functional divergence and amino acid sequence homology, plant terpene synthases (TPS) are systematically classified into seven subfamilies: TPS-a through TPS-h (Aubourg et al., 2002). Among these, the TPS-a subfamily is of particular relevance to sesquiterpenoid biosynthesis (Hendrickson et al., 2024), sesquiterpenoids stand out by featuring complex carbon skeletons with extensive oxidative modifications (Fu et al., 2019; Du et al., 2022), constituting core bioactive components in numerous medicinal species (Wang et al., 2018; Liu et al., 2022). The characteristic volatile profiles of *A. tatarinowii* rhizomes and leaves (Du et al., 2008; Zhang et al., 2015), dominated by structurally diversified sesquiterpenoids (Ye et al., 2006), underpin its chemical defense mechanisms against biotic stressors (Leitner et al., 2005; Jiang et al., 2018; Rahimova et al., 2025).

To elucidate the molecular basis of the characteristic mosquito-repellent odor and its key bioactive terpenoids, this study focused on core volatile sesquiterpenes (Okoli et al., 2021; Ma et al., 2023a). Systematic screening and cloning identified terpene synthase (TPS) family members, with particular attention to the TPS-a subfamily known for sesquiterpene biosynthesis (Han et al., 2022; Jia et al., 2022; Hendrickson et al., 2024). These enzymes feature conserved C-terminal catalytic domains (50–100 kDa) containing Mg²^+^-binding DDXX(D/E) and NSE/DTE motifs, which are essential for substrate ionization (Starks et al., 1997; Yang et al., 2022; Chen et al., 2023).

Headspace solid-phase microextraction coupled with gas chromatography–mass spectrometry (HS-SPME-GC-MS) analysis of leaf volatiles showed that *β*-caryophyllene was the dominant terpenoid in leaves, while the peak detected as *β*-elemene was also abundant (He et al., 2022). Functional characterization suggested that the sesquiterpene synthase AtsTPS2 produces germacrene A, which is detected as *β*-elemene under HS-SPME-GC-MS conditions. Previous studies have shown that *β*-elemene is generally not considered the direct product of sesquiterpene synthases, but is instead formed from germacrene A through a single-step molecular rearrangement under heating or acidic conditions (de Kraker et al., 1998; Zhang et al., 2024). Therefore, the *β*-elemene peak detected under HS-SPME-GC-MS conditions should be regarded as a product formed during analysis rather than the authentic *in planta* product. The catalytic mechanism was further investigated via molecular docking and site-directed alanine mutagenesis (Chen et al., 2025). Transcriptional regulation is a major approach in the regulation of plant terpenoid metabolism, and this has been confirmed in a variety of plants. For instance, LaMYC4 from lavender regulates volatile terpenoid production (Dong et al., 2022), while *Arabidopsis* MYC2 interacts with DELLA proteins to control sesquiterpene synthase expression (Hong et al., 2012). Our study identified AtsMYC5 as a negative regulator of *AtsTPS2* expression. These integrated findings suggest that AtsTPS2-associated volatiles may contribute to the chemical defense system of *A. tatarinowii*, providing new insights into the metabolic regulation and ecological roles of terpenoids in this medicinal plant.

## 2 Results and discussion

### 2.1 Analysis of volatile terpenoid compounds in *Acorus tatarinowii*

**Figure 1:**
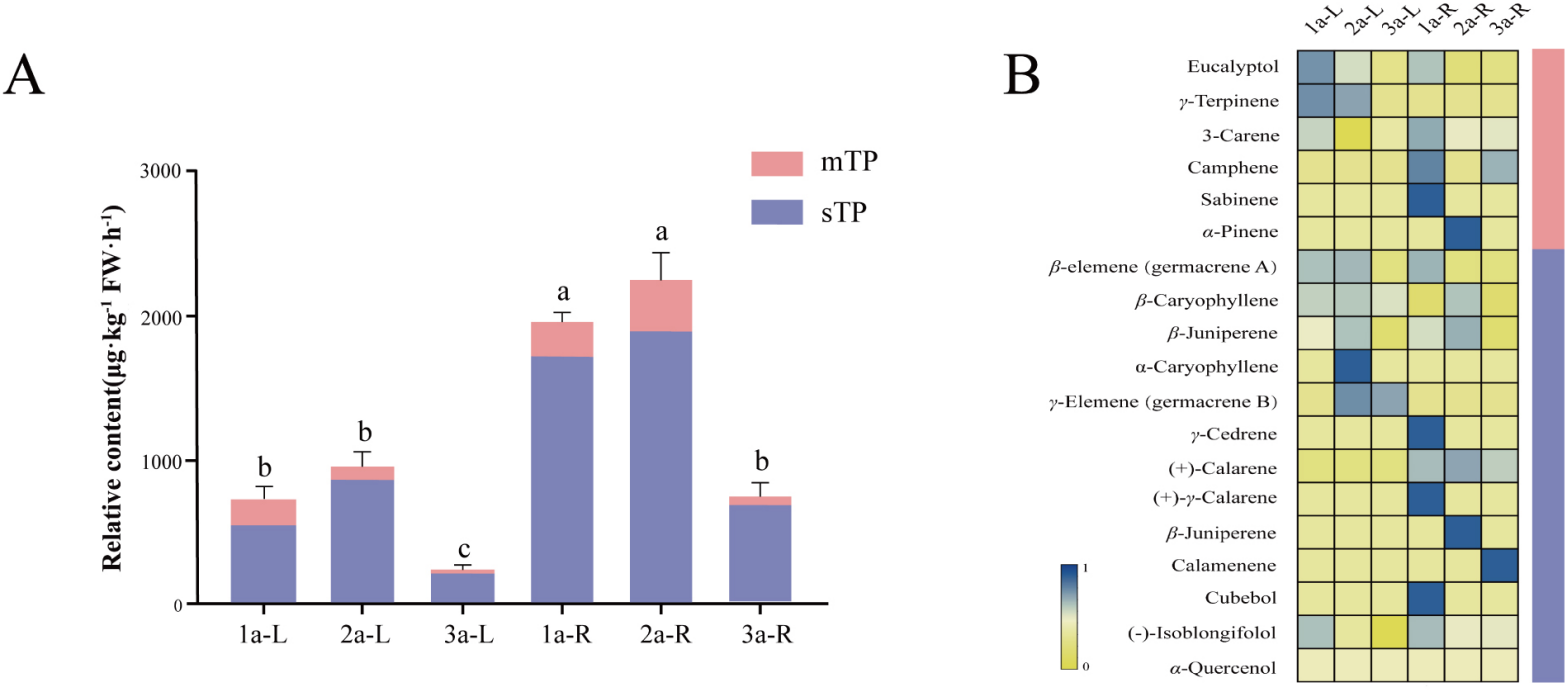
Analysis of volatile terpenoids in the rhizomes and leaves of *A. tatarinowii* of different years by HS-SPME-GC-MS. **(A)** Total content of volatile terpenoids. **(B)** Composition and relative content of individual compounds. 1a is annual, 2a is biennial, 3a is triennial, monoterpenes (mTP), sesquiterpenes (sTP), all samples in each year group had three biological replicates, Internal standard: ethyl decanoate, 200 μg·kg^-^¹ FW·h^-^¹, Data were normalized. The signals labeled as *β*-elemene and *γ*-elemene in the heatmap were later interpreted as germacrene A and germacrene B, respectively, detected under the analytical conditions.

A comparative developmental-stage analysis of terpenoid profiles in the rhizomes and leaves of *A. tatarinowii* was performed by HS-SPME-GC-MS, with ethyl decanoate serving as the internal standard (**Figure 1A**) (**Supplementary Table 1**). Marked differences in terpenoid composition were observed between organs and across age groups. In leaf tissues, *β*-caryophyllene was the dominant terpenoid at all developmental stages. In contrast, germacrene A, which has often been detected as *β*-elemene in previous GC–MS-based studies (de Kraker et al., 1998), showed relatively high abundance only in one- and two-year-old plants. In contrast, rhizomes terpenoid profiles showed pronounced developmental specificity, with substantial variations in composition and concentration. Notably, two-year-old rhizomes exclusively contained *α*-pinene and *β*-juniperene, whereas calamenene was unique to three-year-old rhizomes. Eucalyptol and (-)-Isolongifolol were ubiquitously detected in all six sample groups (**Figure 1B**). Quantitative evaluation further revealed that terpenoid levels were generally higher in rhizomes than in leaves, with biennial plants exhibiting the most intense accumulation, significantly surpassing those of other stages. Given that sesquiterpenoids represent the principal volatile components detected in the rhizomes and leaves of *A. tatarinowii*, with abundant signals including *β*-caryophyllene and germacrene A (which is detected as *β*-elemene), these findings underscore the ontogenetic and organ-specific regulation of terpenoid biosynthesis in this species. We hypothesized that they might contribute to its characteristic mosquito-repellent properties. To test this hypothesis and elucidate the underlying molecular mechanisms, we placed emphasis on the key enzyme in sesquiterpenoid synthesis.

### 2.2 Analysis of TPS gene family in *A. tatarinowii*

**Figure 2:**
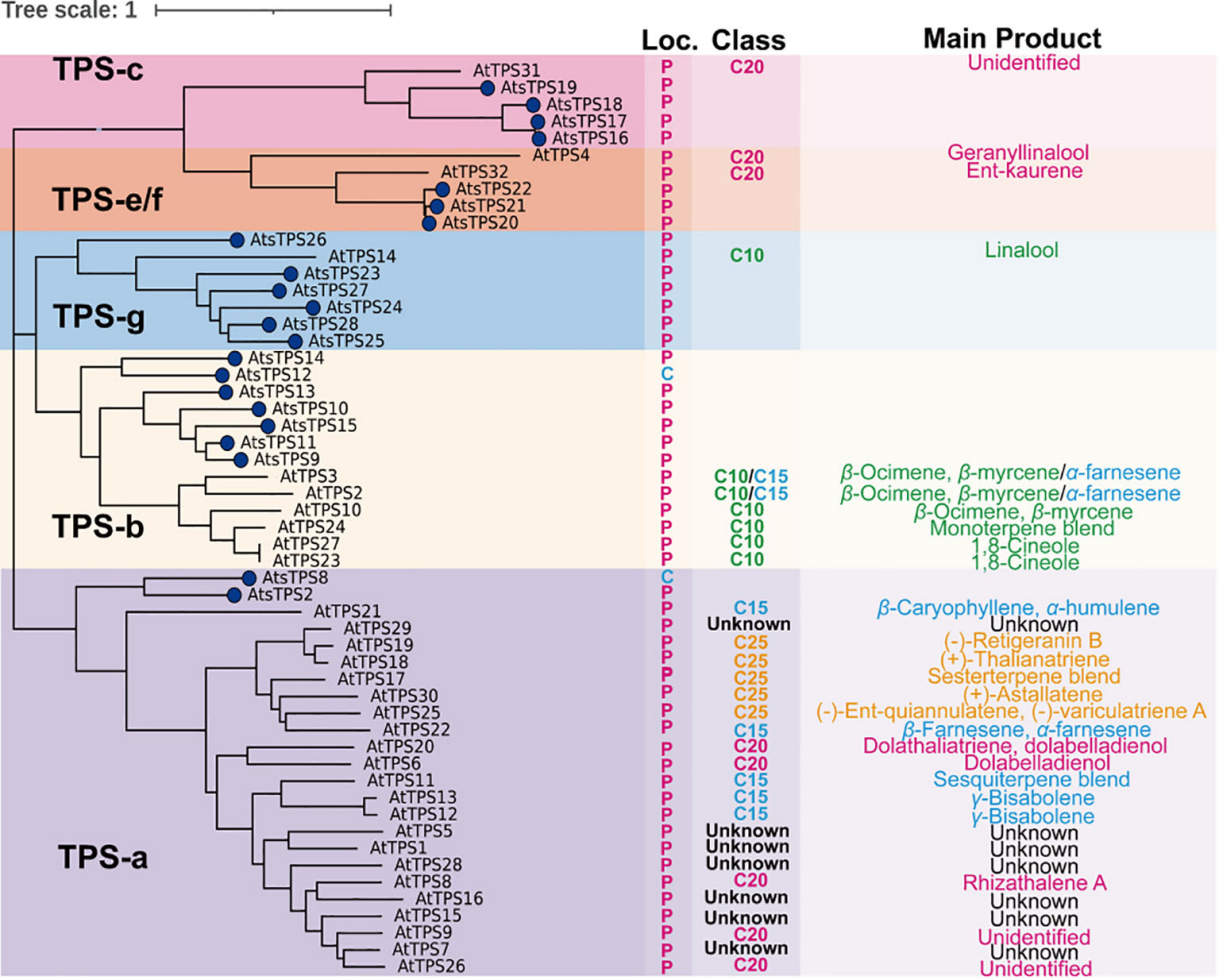
Phylogenetic and functional divergence of terpene synthases in *Acorus tatarinowii* and *Arabidopsis thaliana*. A maximum-likelihood phylogeny of TPS proteins is shown (left), with corresponding subcellular localization, terpenoid class, and dominant enzymatic products (right panels). Major subfamilies are color-coded: TPS-a (purple), TPS-b (yellow), TPS-g (blue), TPS-e/f (orange), TPS-c (red). Localization: Cytoplasm (C, blue), Chloroplast (P, magenta). Terpenoid classes: C_10_ monoterpenes (green), C_15_ sesquiterpenes (blue), C_20_ diterpenes (red), C_25_ sesterterpenes (orange). Sweet flag TPSs (blue) are distinguished from *Arabidopsis thaliana* homologs.

**Figure 3:**
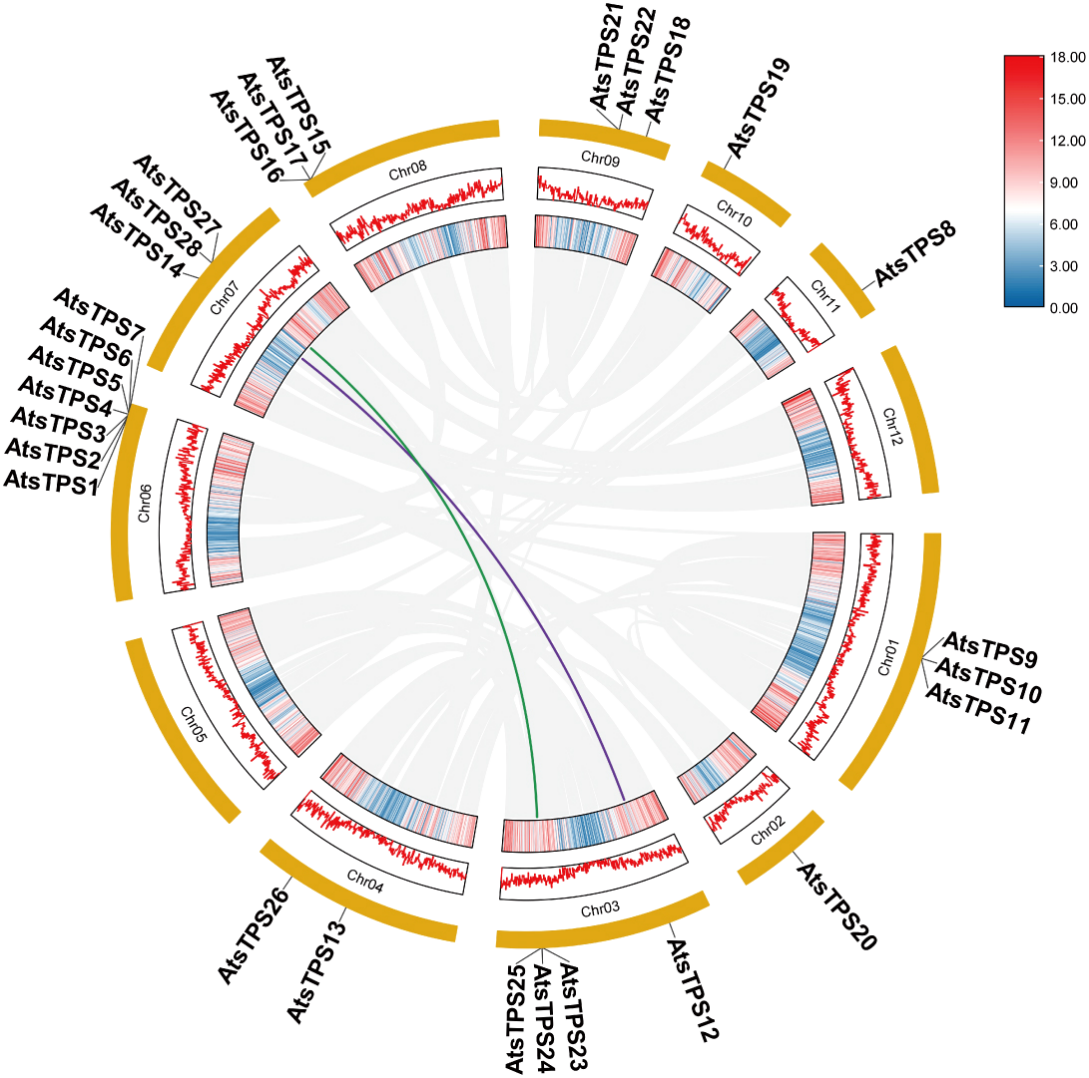
Chromosomal localization, gene density, and synteny analysis of sweet flag TPS genes. Physical positions of TPS genes (vertical ticks) on chromosomes (Chr), with background gradient indicating gene density. Syntenic relationships between TPS loci. Purple lines connect syntenic pairs AtsTPS12 and AtsTPS14; green lines connect AtsTPS23 and AtsTPS28.

HS-SPME-GC-MS analysis confirmed that sesquiterpenes constitute the predominant components in the volatile profiles of rhizomes, and leaves from 1–3-year-old *Acorus tatarinowii* plants, correlating with its characteristic insect-repellent properties. To elucidate the molecular basis of sesquiterpenoid biosynthesis, we reconstructed the phylogeny of the terpene synthase (TPS) gene family, specifically targeting the TPS-a subfamily known to encode sesquiterpene synthases. A total of 28 TPS genes were identified in the *A. tatarinowii* genome. Among them, 22 encoded proteins containing complete TPS domains and were therefore included in the phylogenetic analysis. The remaining six candidates were excluded because their predicted protein sequences were incomplete and lacked key conserved motifs required for reliable phylogenetic placement. The evolutionary tree generated from *A. tatarinowii* and *Arabidopsis thaliana* TPS sequences (**Figure 2**) revealed that TPS-a represents the largest subfamily in *A. tatarinowii* (comprising eight members). Chromosomal localization (**Figure 3**) demonstrated spatial clustering of TPS-a members: *AtsTPS1*–*AtsTPS7* form a tight cluster on chromosome 6, while *AtsTPS8* maps separately to chromosome 11. Synteny analysis identified conserved blocks containing TPS gene pairs between chromosomes 3 and 7, suggesting their origin from segmental duplication events followed by chromosomal translocations. This genomic architecture underpins functional diversification of sesquiterpene synthases and provides a structural framework for coordinated metabolic regulation. To refine phylogenetic resolution within *A. tatarinowii* TPS (*AtsTPSs*), we analyzed the exon-intron architecture and conserved protein motifs of *A. tatarinowii* AtsTPSs using whole-genome annotations (**Supplementary Figure 1**) (Shi et al., 2022). Notably, although TPS-a subfamily members AtsTPS2 and AtsTPS8 exhibit significant divergence in their exon-intron architecture and genomic loci, they both possess an identical set of motifs with the same sequential order. This indicates that while their gene structures have differentiated, the core functional domains remain conserved. The results of the analysis of the physical and chemical properties encoded by the 28 *AtsTPSs* family members of *A. tatarinowii* are shown in **Supplementary Table 2**. The gene IDs and names of the 28 *AtsTPS* family members, along with their orthologs in *Arabidopsis thaliana*, are listed in **Supplementary Table 3**.

### 2.3 *In vitro* functional characterization of *AtsTPS2*

**Figure 4:**
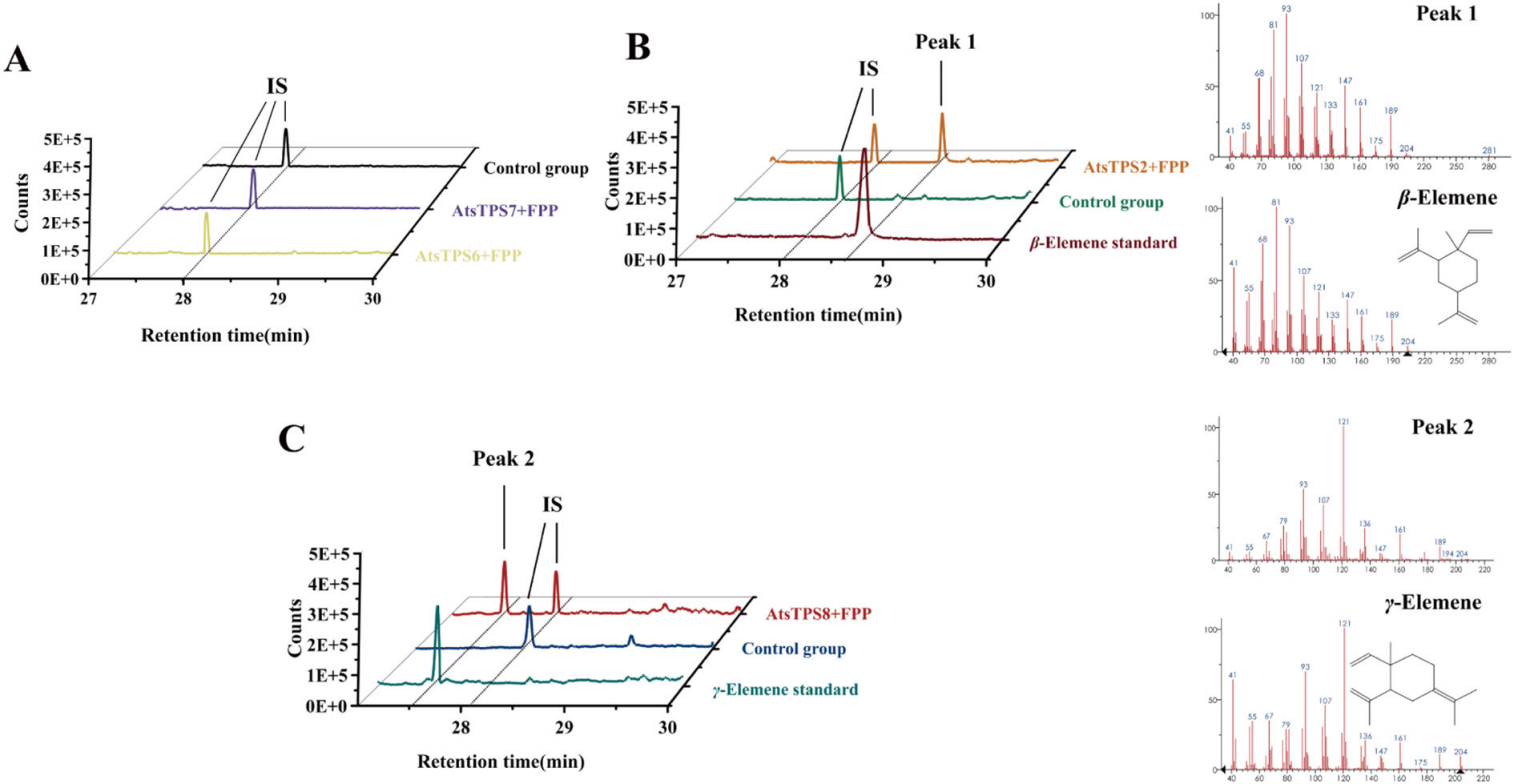
*In vitro* enzymatic product analysis of AtsTPS2, 6, 7, and 8 using FPP as substrate. **(A)** HS-SPME-GC-MS analysis of AtsTPS6 and AtsTPS7 enzymatic assays. No sesquiterpene products were detected (supplemented with ethyl decanoate as internal standard, IS). **(B)** The experimental group (supplemented with ethyl decanoate as an internal standard, IS) showed Peak 1 as the product generated by AtsTPS2 from farnesyl pyrophosphate (FPP). Under HS-SPME-GC-MS conditions, Peak 1 exhibited a fragment ion spectrum and retention time consistent with those of the authentic *β*-elemene standard, but was interpreted as germacrene A detected as *β*-elemene under the analytical conditions. No corresponding peak was detected in the control group. **(C)** The experimental group (supplemented with ethyl decanoate as an internal standard, IS) showed Peak 2 as the product generated by AtsTPS8 from farnesyl pyrophosphate (FPP). Under HS-SPME-GC-MS conditions, Peak 2 exhibited a fragment ion spectrum and retention time consistent with those of the authentic *γ*-elemene standard, but was interpreted as germacrene B detected as *γ*-elemene under the analytical conditions. No corresponding peak was detected in the control group.

In angiosperms, the TPS-a subfamily is primarily responsible for sesquiterpene synthesis; although some members of the TPS-b subfamily can catalyze sesquiterpene formation under certain conditions, the TPS-b subfamily mainly functions as monoterpene synthases. Its sesquiterpene synthase activity is often regarded as a derivative or side activity rather than a representation of the primary function of this subfamily (Wang et al., 2025). Therefore, further analysis of the TPS-a subfamily in *Acorus tatarinowii* was first conducted. Sequence alignment of the eight identified TPS-a family members (*AtsTPS1*-*8*) in *Acorus tatarinowii* revealed that *AtsTPS1*, *2*, *3*, *4*, *6*, *7*, and *8* contain the conserved sesquiterpene synthase catalytic motifs (R/KXR, DDXXD, and NSE/DTE), whereas *AtsTPS5* lacks these motifs entirely (**Supplementary Figure 2**), strongly suggesting it is a non-functional pseudogene. Notably, *AtsTPS1*, *3*, and *4* exhibit significantly shorter amino acid sequences compared to canonical functional sesquiterpene synthases, indicating that they are also likely pseudogenes despite retaining partial catalytic motifs. The pseudogenization of these clustered TPS-a members (*AtsTPS1*, *3*, *4*, *5*) probably results from reduced purifying selection pressure due to functional redundancy within the tandem array on the chromosome. Under such conditions, the probability increases that deleterious mutations—including small insertions or deletions (indels) causing frameshifts or premature stop codons—accumulate in individual gene copies without elimination by natural selection, ultimately leading to loss of function. Consistent with this, heterologous expression of all four *AtsTPS* genes in *E. coli* BL21 using the pMAL-C2X expression system, followed by *in vitro* assays with FPP and HS-SPME-GC-MS analysis, yielded detectable sesquiterpene products exclusively for AtsTPS2 and AtsTPS8. In contrast, no sesquiterpene production was detected for AtsTPS6 and AtsTPS7 (**Figure 4A**), despite the presence of conserved motifs. The most likely cause is that *AtsTPS6* and *AtsTPS7*, located within the tandem repeat region on chromosome 6, accumulated deleterious mutations at critical sites due to relaxed selective pressure, resulting in their loss of function (Zou et al., 2009; Butler et al., 2018). The absence of activity for the remaining members, including *AtsTPS1*, *3*, *4*, *6*, and *7* which possess the conserved motifs but are evidently non-functional (truncated or otherwise compromised), parallels observations in other plant TPS families. Similarly, none of the enzymes produced detectable monoterpenes when supplied with geranyl diphosphate (GPP). Based on mass spectral fragmentation patterns, the product of AtsTPS2 was assigned predominantly as *β*-elemene under HS-SPME-GC-MS conditions (**Figure 4B**), which was formed by thermal Cope rearrangement of germacrene A (de Kraker et al., 1998). Under the same analytical conditions, the product of AtsTPS8 was assigned predominantly as *γ*-elemene (**Figure 4C**), which likewise reflects rearrangement of germacrene B during analysis (Feng et al., 2025). When geranyl diphosphate (GPP) was used as the substrate for enzymatic assays, no detectable monoterpene products were generated, indicating that AtsTPS2 and AtsTPS8 are specific sesquiterpene synthases that only use FPP as a substrate. In addition, although the detected *β*-elemene was generated from germacrene A during analysis, *β*-elemene has been widely studied because of its reported biological activities (Adio, 2009; Li et al., 2010; Govindarajan and Benelli, 2016). We therefore selected AtsTPS2 for further analysis. While Subcellular localization confirmed the cytosolic residence of *AtsTPS2* (**Supplementary Figure 3**), aligning with its predicted role in the cytosolic mevalonate (MVA) pathway for sesquiterpene biosynthesis.

### 2.4 Structural basis for the AtsTPS2 catalytic mechanism

**Figure 5:**
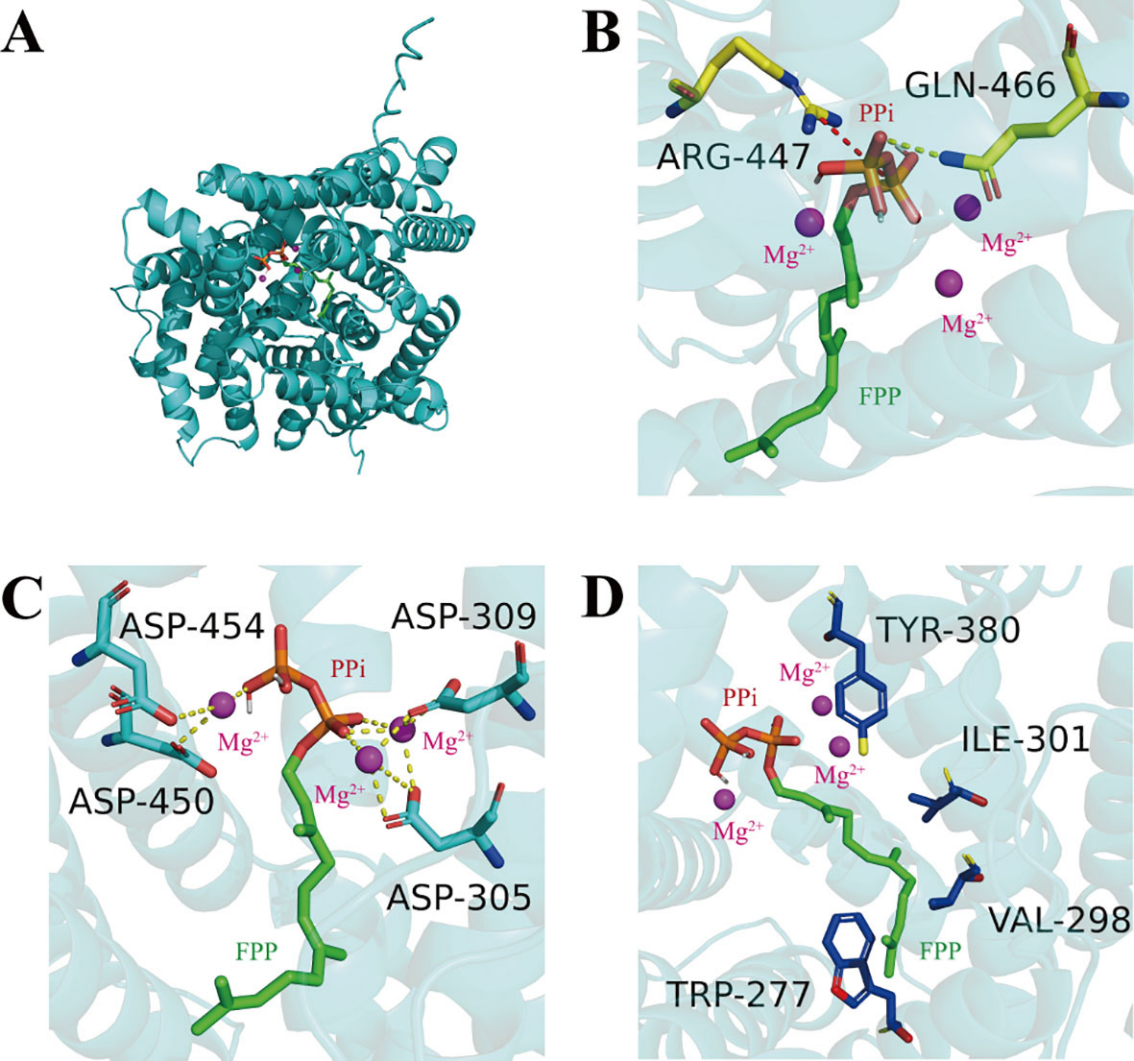
Structural features of AtsTPS2. **(A)** Homology model of AtsTPS2 with molecular docking simulation of substrate FPP. **(B)** Key residues binding the diphosphate moiety (PPi) in AtsTPS2. Salt bridges indicated in red; hydrogen bonds in yellow. **(C)** Key residues coordinating Mg²^+^ ions in AtsTPS2. Metal-ligand interactions indicated in purple. **(D)** Hydrophobic residues interacting with FPP in AtsTPS2.

**Figure 6:**
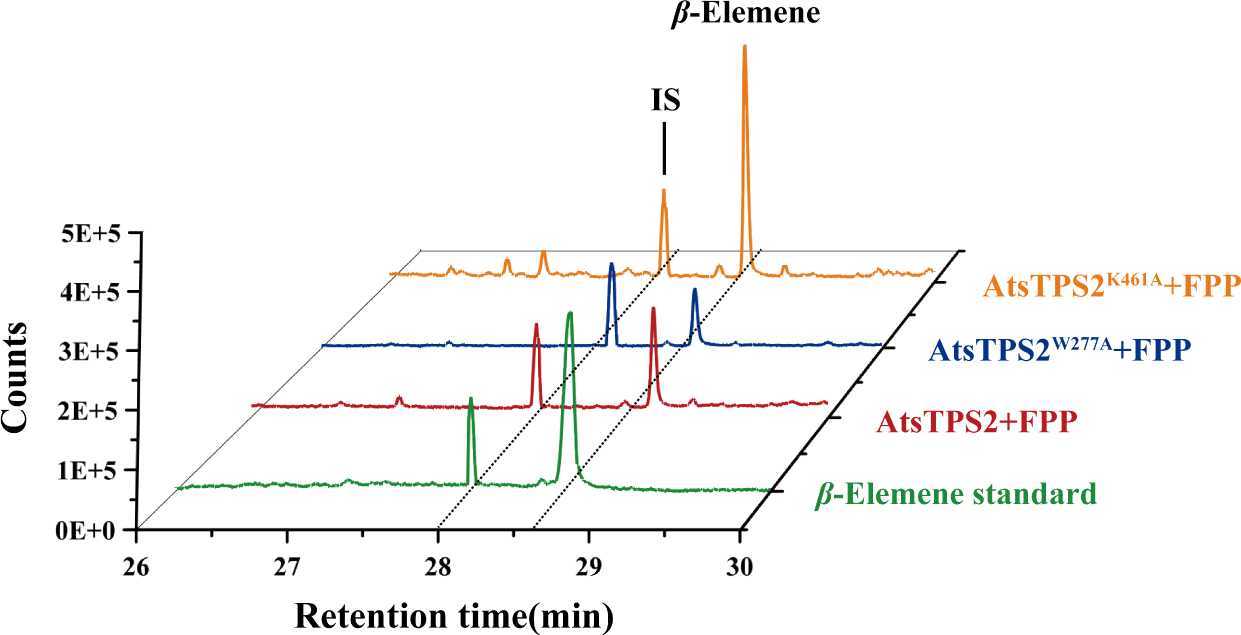
Effects of key-residue mutations in AtsTPS2 on product formation. Ethyl decanoate was used as the internal standard (IS).

Homology modeling of AtsTPS2 using AlphaFold3 (**Figure 5A**) revealed critical interactions within its active site. Two highly conserved residues, Arg447 and Gln466, engage the diphosphate moiety (PPi) via hydrogen bonds (**Figure 5B**). Furthermore, four conserved aspartate residues (Asp305, Asp309, Asp450, Asp454) coordinate three Mg²^+^ ions (**Figure 5C**). The substrate FPP also forms hydrophobic contacts with conserved residues Trp277, Val298, Ile301, and Tyr380, in addition to its interactions with PPi and Mg²^+^ (**Figure 5D**). Site-directed mutagenesis to alanine assessed the functional impact of these residues (**Figure 6**). Compared with the wild-type (WT) enzyme, the W277A mutation reduced the yield of the detected product by 41%, while the K461A mutation increased it by 145% under HS-SPME-GC-MS conditions (**Supplementary Figure 4**). Structural analysis positioned Lys461 at the periphery of the active site, suggesting its potential influence on the conformation of the substrate access channel during FPP entry (**Supplementary Figure 5**). Consistent with previous mutagenesis studies on related terpene synthases, the K461A mutation, reducing steric hindrance at the channel entrance, significantly boosted catalytic activity. Collectively, these results identify Lys461 as a strategic target for modulating AtsTPS2 catalytic efficiency.

### 2.5 *In vivo* functional characterization of *AtsTPS2* by transient transformation

**Figure 7:**
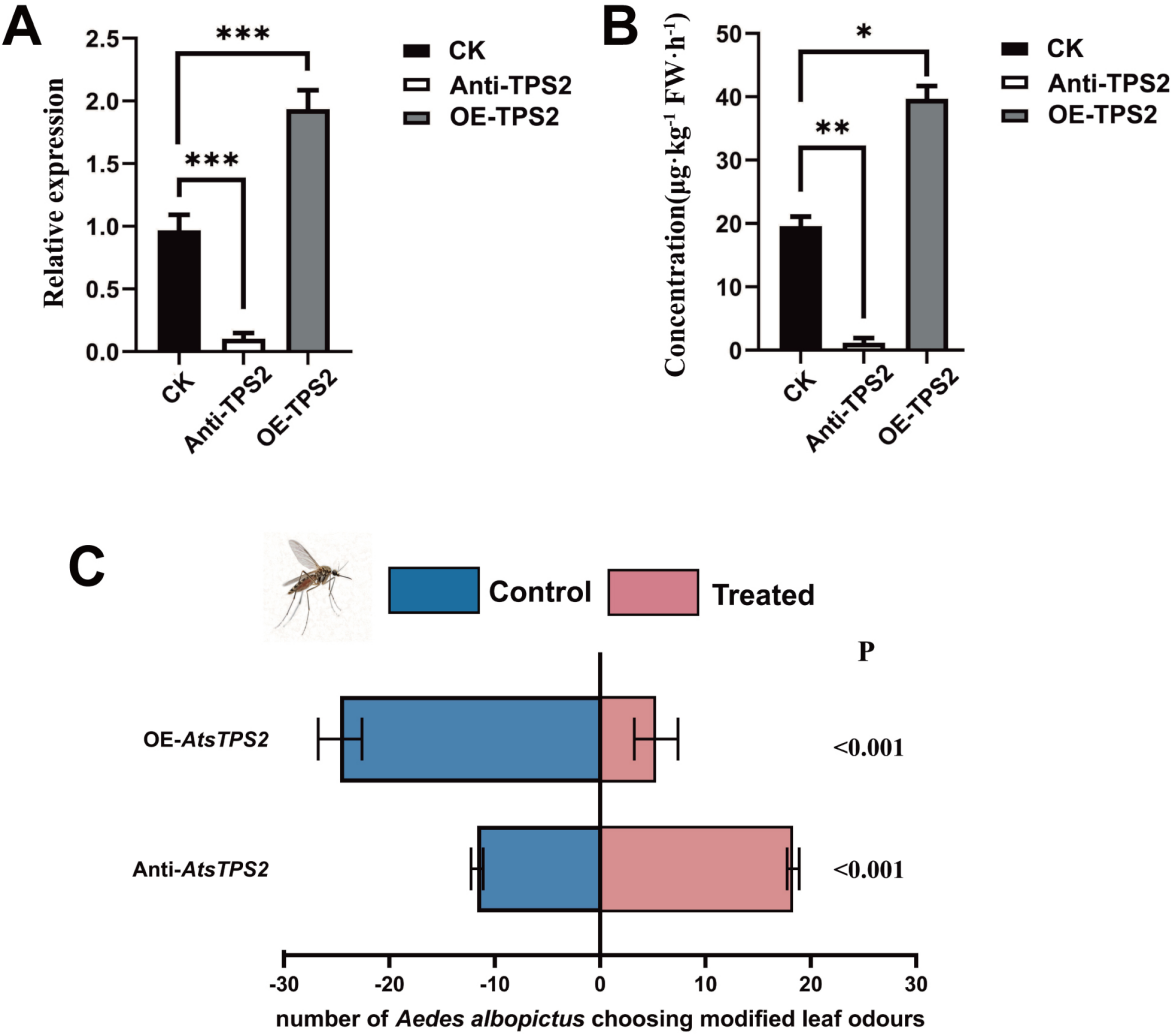
*In planta* functional characterization of AtsTPS2 in *Acorus tatarinowii* and its association with mosquito repellency. **(A)** qRT-PCR analysis of AtsTPS2 transcript levels in wild-type (WT), Anti-*AtsTPS2* (RNAi), and OE-*AtsTPS2* (overexpression) lines, demonstrating significant suppression and overexpression, respectively. **(B)** Relative abundance of germacrene A, detected as *β*-elemene under the analytical conditions, in WT, Anti-*AtsTPS2*, and OE-*AtsTPS2* lines 3 days after treatment. The signal increased markedly in the overexpression lines and decreased significantly in the antisense lines relative to WT. **(C)** Selection of *Acorus tatarinowii* OE-*AtsTPS2* plants and Anti-*AtsTPS2* by *Aedes albopictus*.

To functionally characterize *AtsTPS2 in planta*, we employed antisense RNA-mediated silencing (Anti-*AtsTPS2*) and transient overexpression (OE-*AtsTPS2*) in *Acorus tatarinowii*. qRT-PCR confirmed effective suppression, with Anti-*AtsTPS2* transcript levels reduced by 90%, while OE-*AtsTPS2* lines exhibited a twofold increase in expression relative to WT controls (**Figure 7A**). HS-SPME-GC-MS analysis performed 3 days after treatment showed changes in germacrene A (which is detected as β-elemene) (**Figure 7B**). Compared with the WT, the corresponding peak increased 2.02-fold in the OE-*AtsTPS2* lines, but decreased to 13.2% of the WT level in the Anti-*AtsTPS2* lines.

### 2.6 Changes in *AtsTPS2* expression contribute to mosquito repellency in *A. tatarinowii*

To evaluate the role of *AtsTPS2* in mosquito repellency in *Acorus tatarinowii*, behavioral assays using a Y-tube olfactometer were conducted (**Supplementary Figure 6**). Initial tests compared the repellency of wild-type *A. tatarinowii* leaves against leaves from OE-*AtsTPS2* and Anti-*AtsTPS2*towards *Aedes albopictus*. After a 10-min exposure period (**Figure 7C**), the OE-*AtsTPS2* leaves exhibited significantly higher repellency (83%) compared to WT (65%) and Anti-*AtsTPS2* leaves (59%). To further examine the possible contribution of other co-varying volatiles, we analyzed the volatile profiles of the transiently transformed plants. In addition to germacrene A (which is detected as *β*-elemene), other terpenoid signals also changed in the transformed plants (**Supplementary Figure 7**). In the Anti-*AtsTPS2* lines, most terpenoid peaks showed reduced signal intensity, and germacrene A decreased particularly strongly. In the OE-*AtsTPS2* lines, germacrene A increased significantly, whereas some other peaks, such as *β*-caryophyllene, remained nearly unchanged and more peaks showed reduced signal intensity. Overexpression of *AtsTPS2* may also affect the distribution of shared precursors among different terpene branches (Ma et al., 2023b). Together, these results suggest that mosquito behavioral responses are associated with changes in multiple volatiles, although *AtsTPS2* likely plays an important role.

### 2.7 *In vitro* and *in planta* validation of the interaction between AtsMYCs and *AtsTPS2*

**Figure 8:**
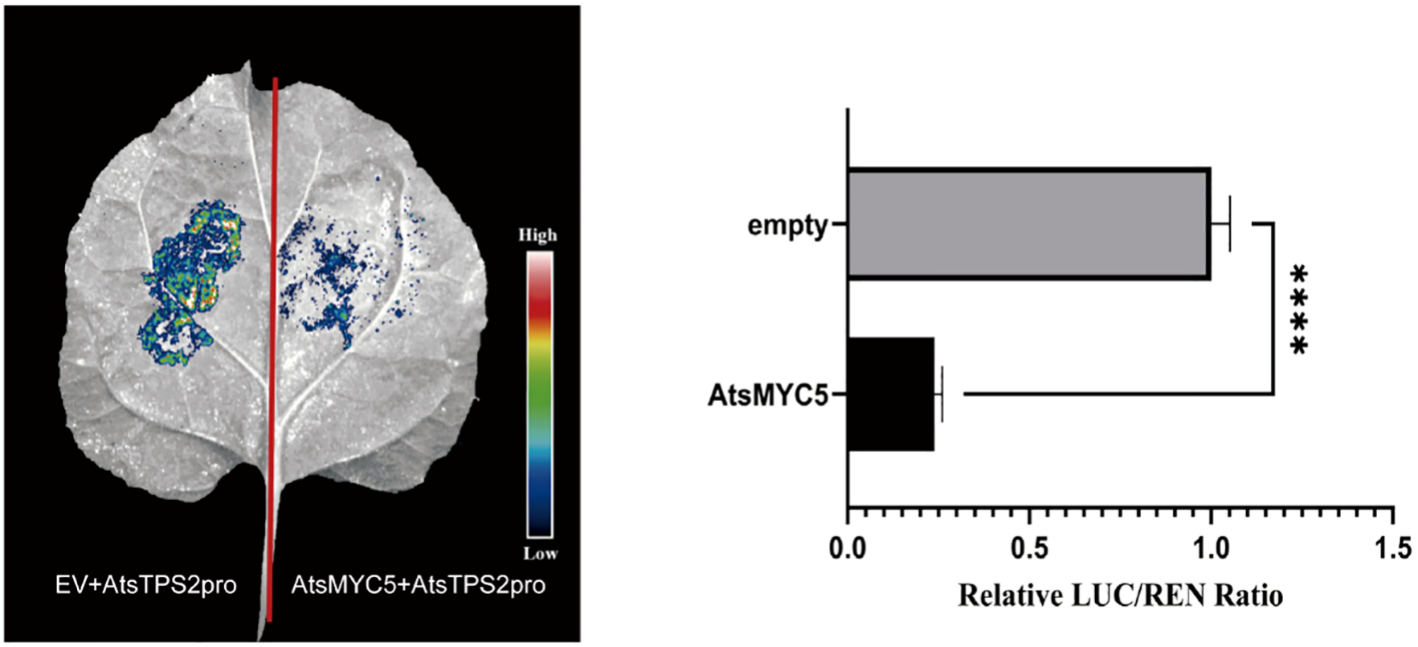
Y1H assay of AtsMYC2/5/6/7 binding to the *AtsTPS2* promoter. Interaction was determined on SD medium lacking leucine in the presence of AbA (AbA 100 ng/mL).

Analysis of the *AtsTPS2* promoter revealed significant enrichment of MYC-binding sites among its cis-acting elements (**Supplementary Figure 8**). Transcriptome data (Guo et al., 2023) indicated co-expression of several AtsMYC transcription factors, and correlation network analysis suggested potential regulatory interactions between these AtsMYCs and *AtsTPS2* (**Supplementary Figure 9**). To experimentally validate the binding of these MYC transcription factors to the promoter, yeast one-hybrid (Y1H) assays were performed. A 120-bp fragment of the AtsTPS2 promoter containing the MYC cis-acting element was used to minimize nonspecific binding (**Supplementary Figure 10**). The assays confirmed that AtsMYC2, AtsMYC5, AtsMYC6, and AtsMYC7 specifically bind to this region, with minimal self-activation detected (**Supplementary Figure 11**). Serial dilution growth assays provided additional confirmation of these direct interactions (**Figure 8**).

**Figure 9:**
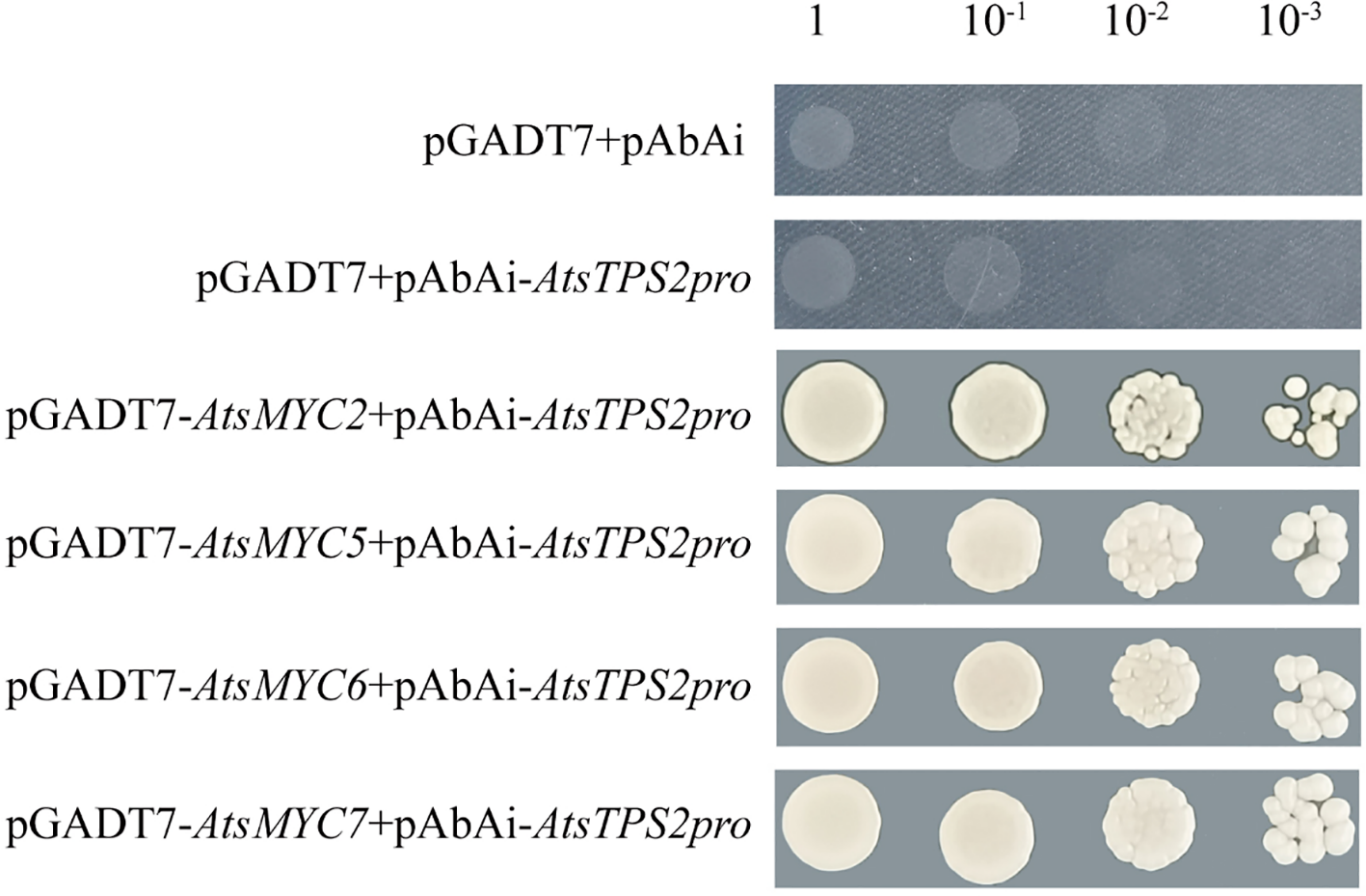
Dual-LUC assay analysis of AtsMYC5 inhibition of *AtsTPS2* promoter activity. Significant differences between the empty vector control and AtsMYC5 effector were detected using the Student’s t-test (****p< 0.0001).

While yeast assays confirmed binding *in vitro*, we next investigated transcriptional regulation *in planta*. Dual-luciferase (Dual-LUC) assays were conducted in *Nicotiana benthamiana* leaves for all four AtsMYCs. These assays employed effector constructs expressing individual AtsMYCs under the CaMV 35S promoter and a reporter construct containing the *AtsTPS2* promoter driving firefly luciferase expression. This functional analysis revealed that only AtsMYC5 significantly suppressed the *AtsTPS2* promoter activity, with a 4.3-fold reduction in the normalized LUC/REN ratio compared to the empty vector control (**Figure 9**). In contrast, AtsMYC2, AtsMYC6, and AtsMYC7 did not exhibit significant regulatory effects on the *AtsTPS2* promoter activity. Consequently, we focused on AtsMYC5 for further validation.

**Figure 10:**
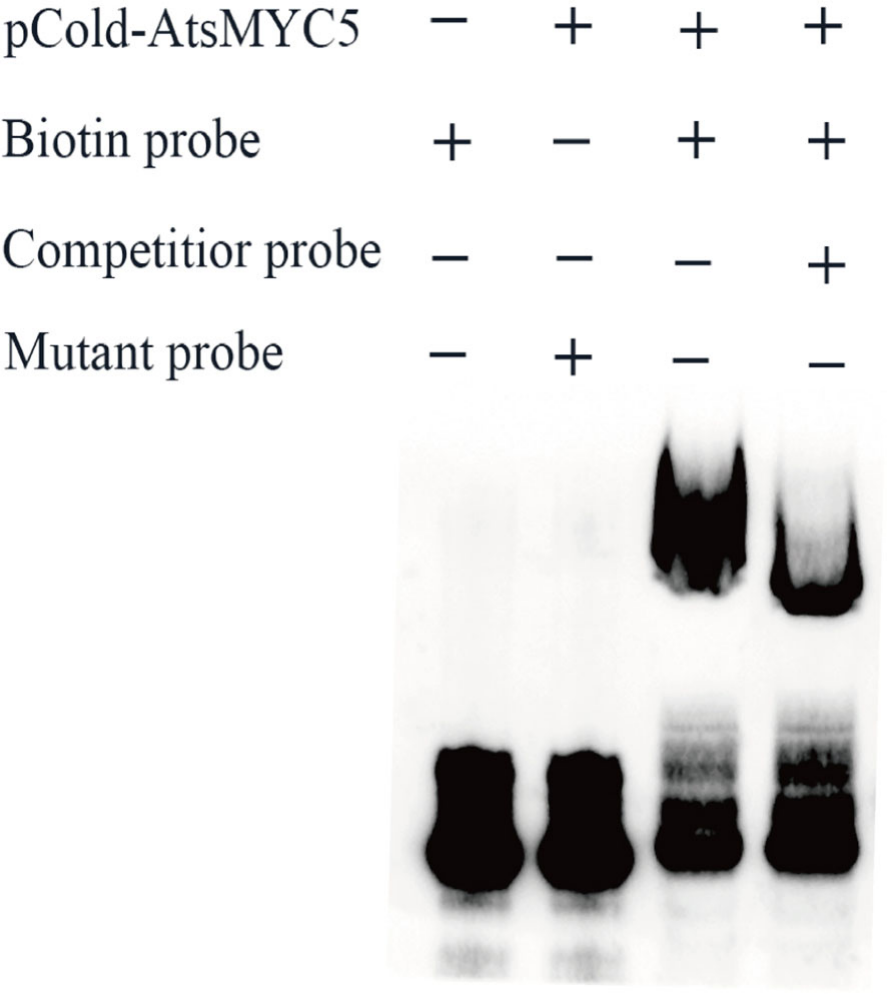
EMSA analysis of AtsMYC5 binding to the *AtsTPS2* promoter probe. Lane 1: Biotin-labeled wild-type probe alone (no protein). Lane 2: pCold-AtsMYC5 protein + biotin-labeled mutant probe. Lane 3: pCold-AtsMYC5 protein + biotin-labeled wild-type probe. Lane 4: pCold-AtsMYC5 protein + biotin-labeled wild-type probe + excess unlabeled wild-type competitor.

Electrophoretic mobility shift assays (EMSA) provided biochemical evidence for the binding specificity of this interaction, showing that AtsMYC5 bound strongly to an E-box cis-element within the *AtsTPS2* promoter. This binding was effectively competed by an unlabeled wild-type probe but not by a mutant version (**Figure 10**). Together, these results indicate that AtsMYC5 acts as a transcriptional repressor of *AtsTPS2* by directly binding to its promoter.

## 3 Conclusions

Genome-wide analysis identified TPS-a subfamily members in *Acorus tatarinowii*, with a gene cluster on chromosome 6, while AtsTPS8 is located separately on chromosome 11. Their evolutionary history reflects segmental duplication and translocation events. Functional assessment revealed that only *AtsTPS2* and *AtsTPS8* encode active sesquiterpene synthases; *AtsTPS1*, *3*, *4*, and *5* are nonfunctional pseudogenes, likely arising from relaxed selective pressure within their tandem array. Under HS-SPME-GC-MS conditions, AtsTPS2 produced germacrene A (which is detected as *β*-elemene), whereas AtsTPS8 produced germacrene B (which is detected as *γ*-elemene). The cytosolic localization of AtsTPS2 is consistent with a role in the MVA pathway. We further identified a highly active AtsTPS2 mutant, K461A, which increased the yield of the corresponding sesquiterpene product by 145% in the *in vitro* assay. *In planta* analysis further showed that overexpression of *AtsTPS2* increased the accumulation of the corresponding sesquiterpene product by 2.02-fold, whereas antisense suppression reduced it to 13.2% of the wild-type level. Transiently transformed lines overexpressing *AtsTPS2* exhibited 83% repellency against *Aedes albopictus*, significantly exceeding the wild-type level (65%).

The negative regulation of AtsTPS2 by AtsMYC5 was experimentally validated through Y1H, dual-LUC, and EMSA. Collectively, these findings highlight *A. tatarinowii* as a rich source of bioactive sesquiterpenes and delineate the genetic and biochemical basis of sesquiterpene-mediated insect resistance. Our results show that AtsTPS2 is associated with sesquiterpene production related to mosquito repellency. Its expression is negatively regulated by AtsMYC5.

## 4 Experimental methods

### 4.1 Plant materials

Both *A. tatarinowii* and *Nicotiana benthamiana* were cultivated in controlled artificial climate chambers (25°C, 16h light/8h dark cycle) under optimized PAR and humidity conditions to ensure standardized physiological status and secondary metabolite production.

### 4.2 HS-SPME-GC-MS analysis of volatile terpene components of *A. tatarinowii*

For volatile analysis, samples were collected from *A. tatarinowii* plants of different ages (one-, two-, and three-year-old plants). Fully expanded mature leaves with uniform growth status and rhizome segments from the middle portion of the rhizome were harvested. All samples were collected at the same time of day. Volatile terpenes in *A. tatarinowii* were analyzed by HS-SPME-GC-MS using a 100 μm PDMS fiber for 1-hour adsorption at room temperature, followed by separation on an HP-5MS column (30m × 0.25mm × 0.25 μm) with helium carrier gas at 1 mL/min under an oven program of 60°C (hold 6min), ramped at 5°C/min to 300°C (hold 5min). For qualitative identification of the enzymatic products, the injector temperature was set at 250°C in splitless mode. The same injector temperature was used for all quantitative comparisons to ensure optimal detection sensitivity (Calvert et al., 2002). MS detection employed electron ionization (70 eV) with ion source at 230 °C, transfer line at 280°C, quadrupole at 150°C, and scanning range of m/z 50-400. Compound identification utilized the NIST 2011 library and literature comparisons (Yahyaa et al., 2015; Xu et al., 2019; Li et al., 2021, Li et al., 2024). Each treatment included three biological replicates, and each biological replicate was analyzed with three technical replicates. Quantification was based on ethyl decanoate internal standard (200 μg·kg^-^¹ FW·h^-^¹) using peak area, with volatile content expressed as μg·kg^-^¹ FW·h^-^¹. Source: *β*-elemene Standard (GC ≥ 98%) was purchased from Yuanye Biotechnology (Product Code: B23134-20mg), *γ*-elemene Standard (GC ≥ 98%) was purchased from Weikeqi Biotechnology (Product Code: WKQ-0007168-20mg).

### 4.3 Computational analysis and alanine mutation

The three-dimensional structure of AtsTPS2 was predicted using AlphaFold3. Subsequent molecular docking simulations with AutoDock Vina (v1.2.7) first positioned Mg²^+^ within the AtsTPS2 model, followed by energy minimization to optimize the complex, then docked the substrate farnesyl diphosphate (FPP) to generate AtsTPS2-FPP complexes. Structural analysis was performed using PyMOL. Site-directed mutagenesis substituting W277 and K461 with alanine was conducted via whole-plasmid PCR: initial denaturation (98°C, 3min); 25 cycles of 98°C (15 s), 54°C (15 s), 72°C (100 s); final extension (72°C, 10min). PCR products were digested with DpnI (30min) to remove methylated parental DNA, purified, and transformed into *E. coli* BL21 competent cells.

### 4.4 Bioinformatics analysis

Genome-wide *Acorus tatarinowii* protein sequences retrieved via TBtools were screened for PF01397 (TPS) and PF03936 (TPS_C) Pfam domains to identify putative terpene synthase genes (*AtsTPSs*), with essential domain presence validated using SMART; ExPASy ProtParam predicted basic protein characteristics (MW, pI). Evolutionary relationships were investigated by aligning *AtsTPS* sequences with *A. tatarinowii* and *Arabidopsis thaliana* homologs in MEGA, followed by Maximum Likelihood phylogenetic reconstruction (ClustalW alignment, 1000 bootstrap replicates). TBtools visualized *AtsTPS* gene structure (exon-intron) and chromosomal localization; the MEME Suite identified conserved protein motifs. Putative promoter regions (2000 bp upstream of start codons) were analyzed for cis-acting regulatory elements (stress responses, hormone signaling, light regulation) using PlantCARE.

### 4.5 Quantitative real-time PCR analysis

Total RNA was extracted from samples using the Plant RNA Extraction Kit V1.5. Reverse transcription to cDNA was performed with the MonaMonScript™ RTIII SuperMix with dsDNase. Quantitative PCR (qPCR) amplification was carried out under the following conditions: initial denaturation at 95°C for 3 min; 40 cycles of denaturation at 95°C for 10s, annealing/extension at 60°C for 30s; followed by melt curve analysis (95°C for 15 s, 65°C for 5s, and 95°C for 5s). Three biological replicates were included, and relative gene expression levels were calculated using the 2^−ΔΔCt^ method (Livak and Schmittgen, 2001).

### 4.6 Heterologous expression and enzymatic activity of *AtsTPS2*, *AtsTPS6*, *AtsTPS7*, and *AtsTPS8*

The full-length AtsTPS2, *AtsTPS6, AtsTPS7, and AtsTPS8* sequence were amplified via PCR using primers with XbaI and PstI restriction sites and cloned into the pMAL-C2X vector. The recombinant plasmid (pMAL-C2X-*AtsTPS2*) was transformed into *E. coli* BL21 cells. Protein expression was induced overnight at 16°C. Crude protein extracts were purified using MBP affinity chromatography (dextrin bead 6FF resin). Enzymatic activity was assayed in a 1 mL reaction mixture consisting of 500 μL crude protein extract, 100 μL 50 mM Tris-HCl (pH 7.5), 20 μL each of 10 mM MgCl_2_ and MnCl_2_, 1 μL FPP or GPP, and 359 μL ddH_2_O. After incubation at 30°C for 30 min, volatiles were extracted at room temperature (1 h) and analyzed via HS-SPME-GC-MS (method as per Section 4.2) to identify enzymatic products.

### 4.7 Subcellular localization analysis

AtsTPS2 was amplified with gene-specific primers and cloned into the pCAMBIA1300-GFP vector, followed by endotoxin-free plasmid preparation using an EndoFree Plasmid Midi Kit. Transient transfection into *Arabidopsis thaliana* protoplasts was performed using established protocols, with subsequent laser confocal microscopy imaging to track GFP signal subcellular localization.

### 4.8 Y-tube olfactometer experiment

Behavioral responses of *Aedes albopictus* mosquitoes (laboratory colony, maintained at 27 °C/80% RH on 10% sucrose) were evaluated using a Y-tube olfactometer (Dicke et al., 1990). Volatiles delivered via airstream recorded insect choices to determine attraction, repulsion, or neutrality. Groups of 30 mosquitoes per replicate (4-h starved) were tested with wild-type *Acorus tatarinowii* leaves as control versus either OE-*AtsTPS2* or Anti-*AtsTPS2* leaves. All assays included three biological replicates.

### 4.9 Analysis of the interaction of AtsMYCs with *AtsTPS2*

FPKM values for *AtsTPS2* and *AtsMYCs* were derived from transcriptome data. Pearson correlation coefficients between gene pairs were calculated using R, retaining pairs with P < 0.05 and correlation coefficient > 0.6. Statistically significant co-expressed gene pairs were visualized as a network using Cytoscape to infer regulatory interactions.

### 4.10 Transcriptional regulation analysis

- Yeast One-Hybrid (Y1H) Assay: A 120-bp *AtsTPS2* promoter fragment containing MYC motifs was cloned into pAbAi. Bait yeast strains (Y1HGold) were generated by integrating linearized pAbAi-*AtsTPS2pro*. The minimal inhibitory concentration of Aureobasidin A (AbA) preventing bait autoactivation was determined in SD/-Ura. *AtsMYC* genes cloned into pGADT7 were co-transformed into bait strains. Interactions were assessed by spotting serial dilutions onto SD/-Leu + AbA, followed by incubation at 28°C for 72h.
- Dual Luciferase (Dual-LUC) Assay: The *AtsTPS2* promoter was fused to Firefly LUC in pGreenII0800. *AtsMYCs* were cloned into pGreenII62-SK. Recombinants were co-transformed into *Agrobacterium tumefaciens* GV3101 (p19). Cultures (OD_600_ = 0.6–0.8) were infiltrated (reporter:effector = 1:9) into abaxial leaf surfaces of 4-week-old *N. benthamiana*. Firefly LUC activity was measured 48–72 h post-infiltration and normalized to co-expressed Renilla LUC activity.
- Electrophoretic Mobility Shift Assay (EMSA): Recombinant AtsMYC5 was expressed in *E. coli* Rosetta (DE3) harboring pCold-*AtsMYC5*. Binding reactions used biotin-labeled *AtsTPS2* promoter oligonucleotides and unlabeled competitors, analyzed via chemiluminescence (Biotin EMSA Kit).

Note: All assays included three biological replicates.

### 4.11 *AtsTPS2* transient transformation to *Acorus tatarinowii* leaves

Construction of OE-*AtsTPS2* (overexpression) Plants: The coding sequence of *AtsTPS2* was cloned into the pCAMBIA1305 vector. The resulting recombinant plasmid was introduced into *Agrobacterium tumefaciens* strain GV3101 (harboring the p19 plasmid) via the freeze-thaw method. For the negative control, *Agrobacterium* carrying the empty pCAMBIA1305 vector was prepared and processed in parallel. Bacterial cells were pelleted and resuspended in agroinfiltration buffer to an OD_600_ of 0.6. After incubation at room temperature for 3 hours, the bacterial suspensions were infiltrated into the leaves of *Acorus tatarinowii*. Following infiltration, the plants were maintained in darkness at 25 ± 1°C for 48 hours, and then transferred to a 16h light/8h dark photoperiod for an additional 24 hours. The treated leaves were subsequently collected for analysis.

Construction of Anti-*AtsTPS2* (RNAi) Plants: Three gene-specific antisense RNA oligonucleotides for *AtsTPS2* were designed using the Sfold web server (OutputforSoligo, Job15759). Uniform, healthy leaves of *A. tatarinowii* were selected and immersed in a 50 μmol/L solution of these primers. A control set was soaked in distilled water. The treatment duration was 3d, after which leaf samples were collected.

## Data Availability

The data analyzed in this study is subject to the following licenses/restrictions: We state the restrictions that apply to the dataset as follows: the dataset is only available for non-commercial research purposes and requires a formal application to the corresponding author for access. Requests to access these datasets should be directed to hhfan0551@126.com.

## Supplementary material

Supplementary Figure 1 Conserved motif and gene structure analysis of *AtsTPSs* genes family. The conserved motifs of *AtsTPSs*. Ten conserved motifs were predicted by the MEME and displayed by different colored boxes. Exon/intron distribution of *AtsTPSs*. The green module represents the UTR non-translation area. The yellow module represents the CDS translation area.

Supplementary Figure 2 Sequence alignment of the eight identified TPS-a family members (AtsTPS1-8) in *Acorus tatarinowii.* The motif in the red box is R/KXR, the motif in the green box is DDXXD, and the motif in the blue box is NSE/DTE.

Supplementary Figure 3 Subcellular localization of AtsTPS2 in *Arabidopsis thaliana* protoplasts. Scale bars indicate 10 µm.

Supplementary Figure 4 Effects of key-residue mutations in AtsTPS2 on the formation of the corresponding sesquiterpene product. The experiments were repeated three times, and the error bar represented the standard deviation of the mean. Significant differences between each group were detected using the Student’s t-test (**p* < 0.05, ***p* < 0.01).

Supplementary Figure 5 Comparison of substrate channel structures between mutant K461A and WT.

Supplementary Figure 6 Schematic diagram of Y-tube olfactometer.

Supplementary Figure 7 HS-SPME-GC-MS peak profiles of volatile terpenoids in WT, Anti-*AtsTPS2*, and OE-*AtsTPS2* lines. WT is shown in blue, Anti-*AtsTPS2* in green, and *OE-AtsTPS2* in red. (A) Representative HS-SPME-GC-MS peak profiles of monoterpenes in the three lines. Peaks labeled in panel A are: 1, eucalyptol; 2, *γ*-terpinene. Other volatile signals in this retention time range are not labeled. (B) Representative HS-SPME-GC-MS peak profiles of sesquiterpenes in the three lines. Peaks labeled in panel B are: 3, germacrene B (detected as *γ*-elemene); 4, germacrene A (detected as *β*-elemene); 5, *β*-caryophyllene; 6, (−)-isolongifolol; 7, juniperene; and 8, *α*-caryophyllene.

Supplementary Figure 8 Cis-acting elements and functions of the *AtsTPSs* promoter.

Supplementary Figure 9 Co-expression network of *AtsTPS2* gene and AtsMYC transcription factor in *A. tatarinowii.*

Supplementary Figure 10 120 bp fragment located from positions -1100 to -980 relative to the transcription start site, TSS. Yellow squares represent MYC-binding site, and the red boxes contain the selected fragment.

Supplementary Figure 11 *AtsTPS2* promoter schematic diagram of segmental self-activation.

Supplementary Table 1 Detailed quantitative data of each volatile terpenoid in all samples.

Supplementary Table 2 Information of *AtsTPSs* gene family members of *A. tatarinowii.*

Supplementary Table 3 Terpene Synthase (TPS) Gene IDs and Names of *Acorus tatarinowii* and *Arabidopsis thaliana.*
