# Supplementary material for: Catalytic mechanism and transcriptional regulation of AtsTPS2 involved in germacrene A biosynthesis in Acorus tatarinowii
Source: Front Plant Sci. 2026 Apr 29;17:1761957. doi: 10.3389/fpls.2026.1761957 (PMC13168051; doi:10.3389/fpls.2026.1761957)
Supplement: Supplementary Figure 1 — Conserved motif and gene structure analysis of AtsTPSs genes family. The conserved motifs of AtsTPSs. Ten conserved motifs were predicted by the MEME and displayed by different colored boxes. Exon/intron distribution of AtsTPSs. The green module represents the UTR non-translation area. The yellow module represents the CDS translation area. [file DataSheet1.docx]

# Supplementary Figures


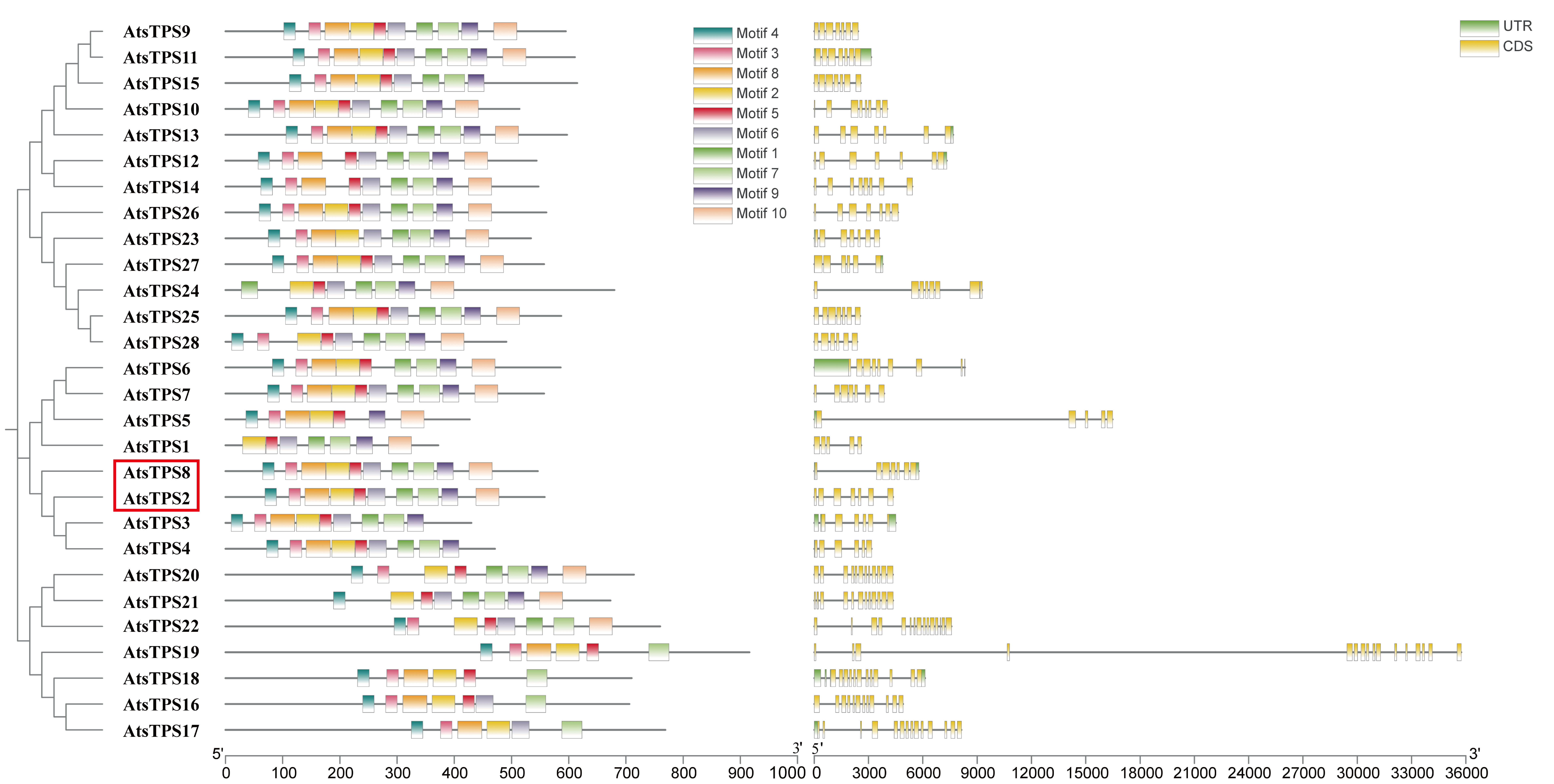


**Supplementary Figure 1.** Conserved motif and gene structure analysis of *AtsTPSs* genes family. The conserved motifs of *AtsTPSs*. Ten conserved motifs were predicted by the MEME and displayed by different colored boxes. Exon/intron distribution of *AtsTPSs*. The green module represents the UTR non-translation area. The yellow module represents the CDS translation area.


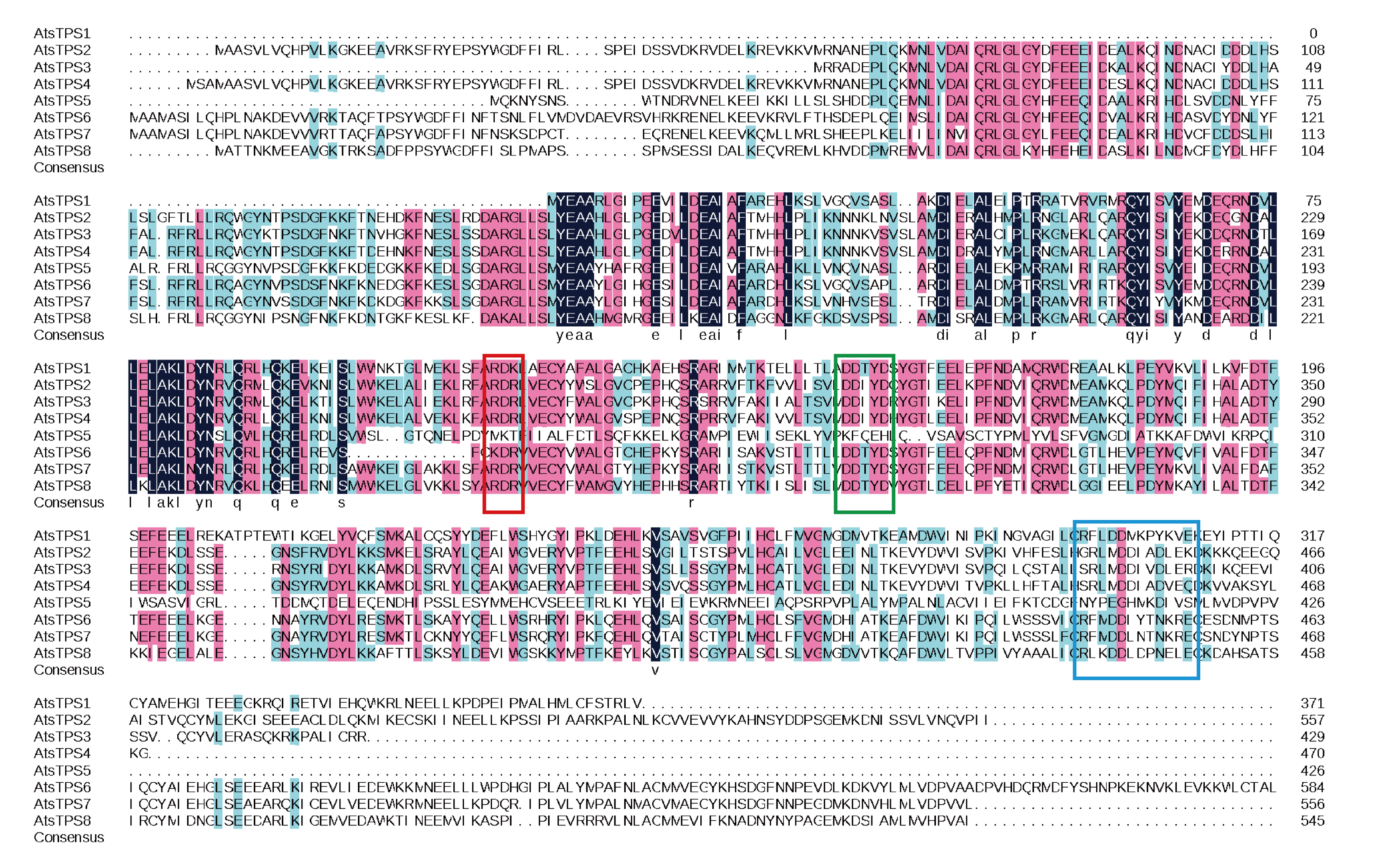


**Supplementary Figure 2.** Sequence alignment of the eight identified TPS-a family members (AtsTPS1-8) in Acorus tatarinowii. The motif in the red box is R/KXR, the motif in the green box is DDXXD, and the motif in the blue box is NSE/DTE


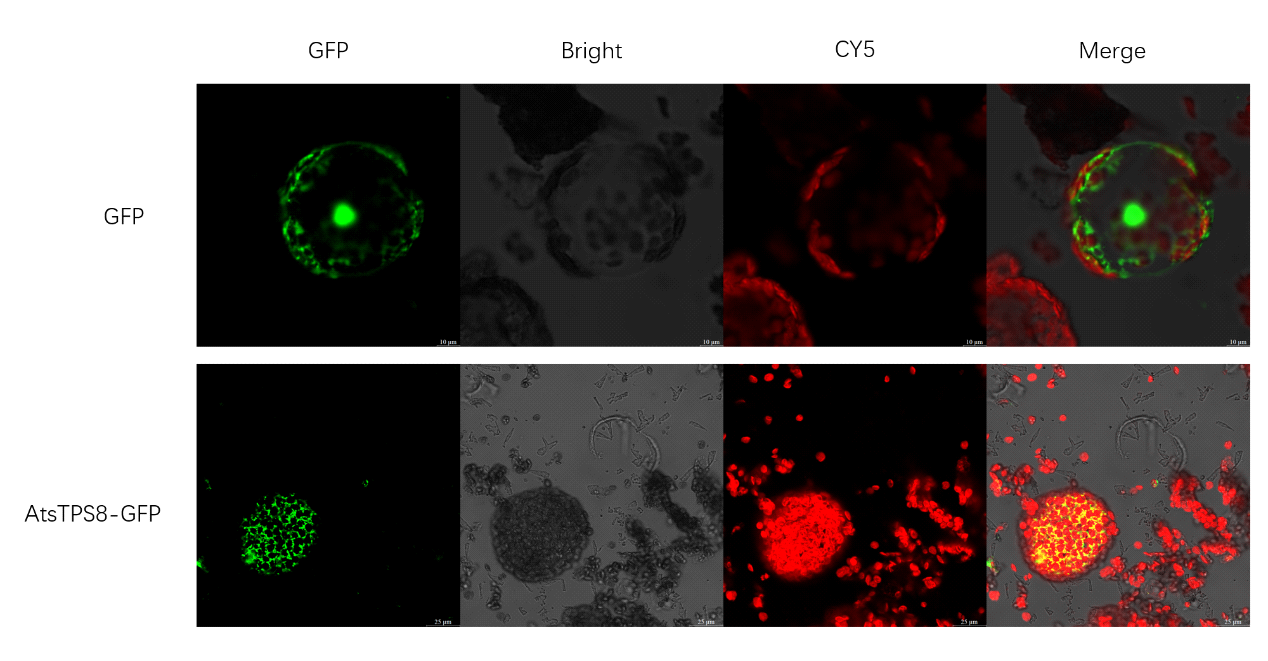


**Supplementary Figure 3.** Subcellular localization of AtsTPS2 in Arabidopsis thaliana protoplasts. Scale bars indicate 10 µm.


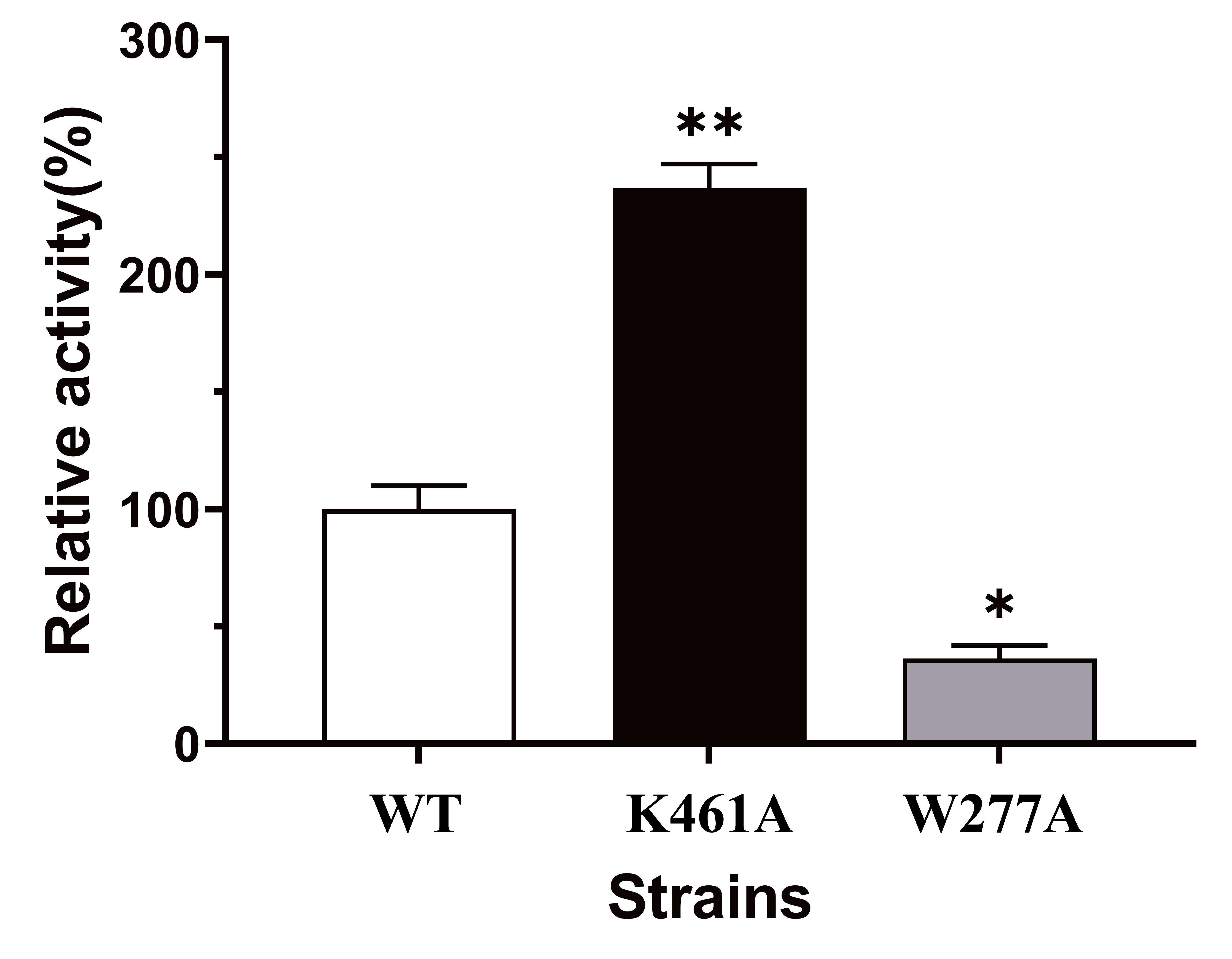


**Supplementary Figure 4.** Effects of key-residue mutations in AtsTPS2 on the formation of the corresponding sesquiterpene product. The experiments were repeated three times, and the error bar represented the standard deviation of the mean. Significant differences between each group were detected using the Student's t-test (**p* < 0.05, ***p* < 0.01).


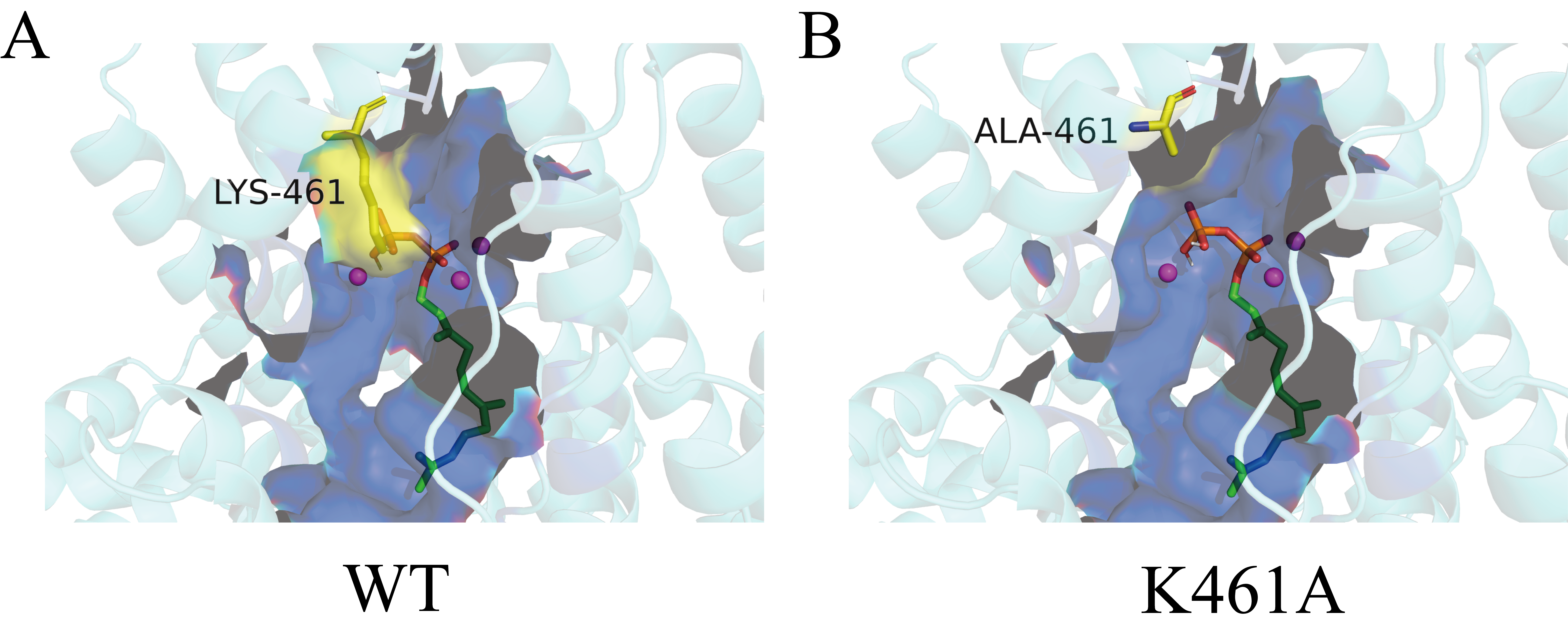


**Supplementary Figure 5.** Comparison of substrate channel structures between mutant K461A and WT.


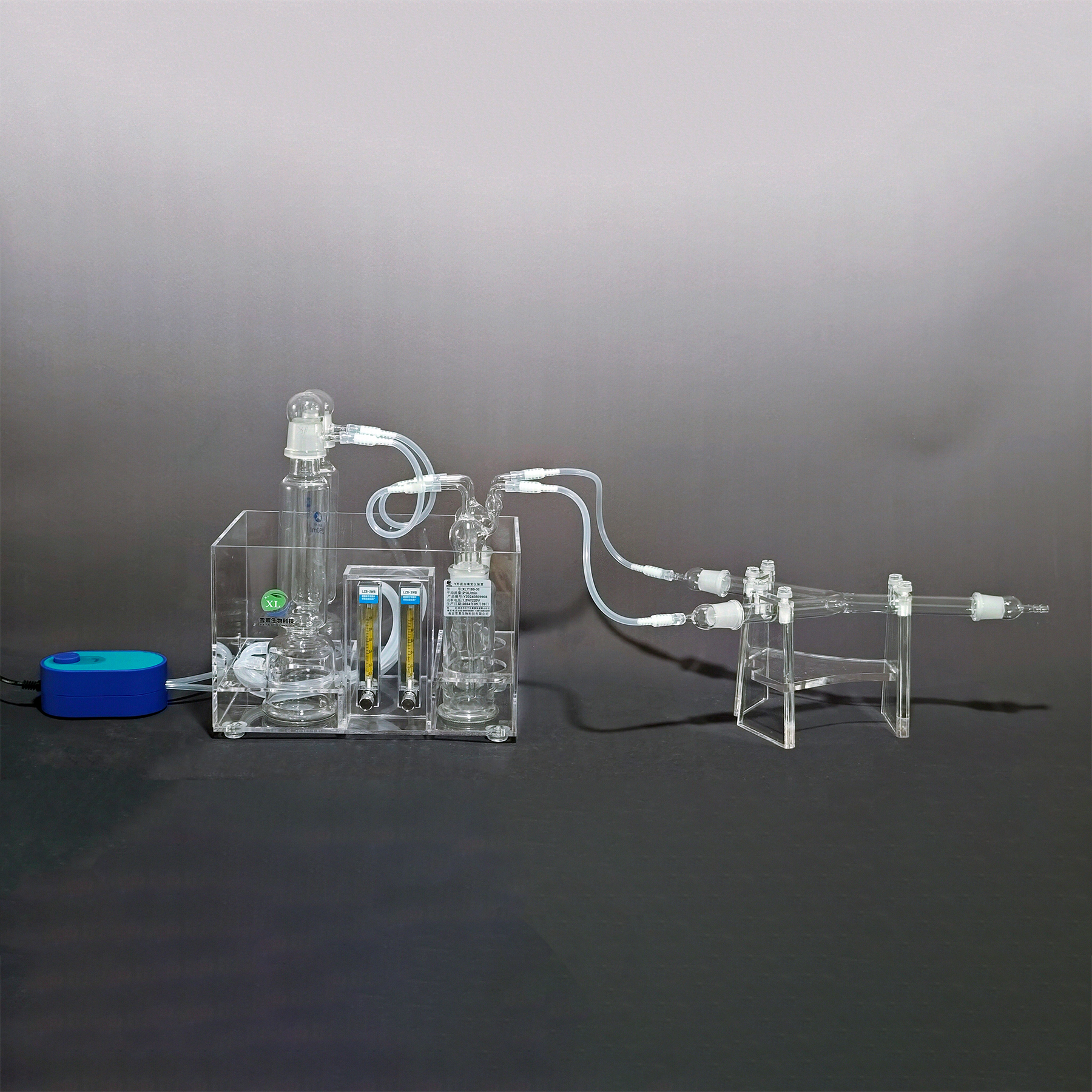


**Supplementary Figure 6.** Schematic diagram of Y-tube olfactometer.


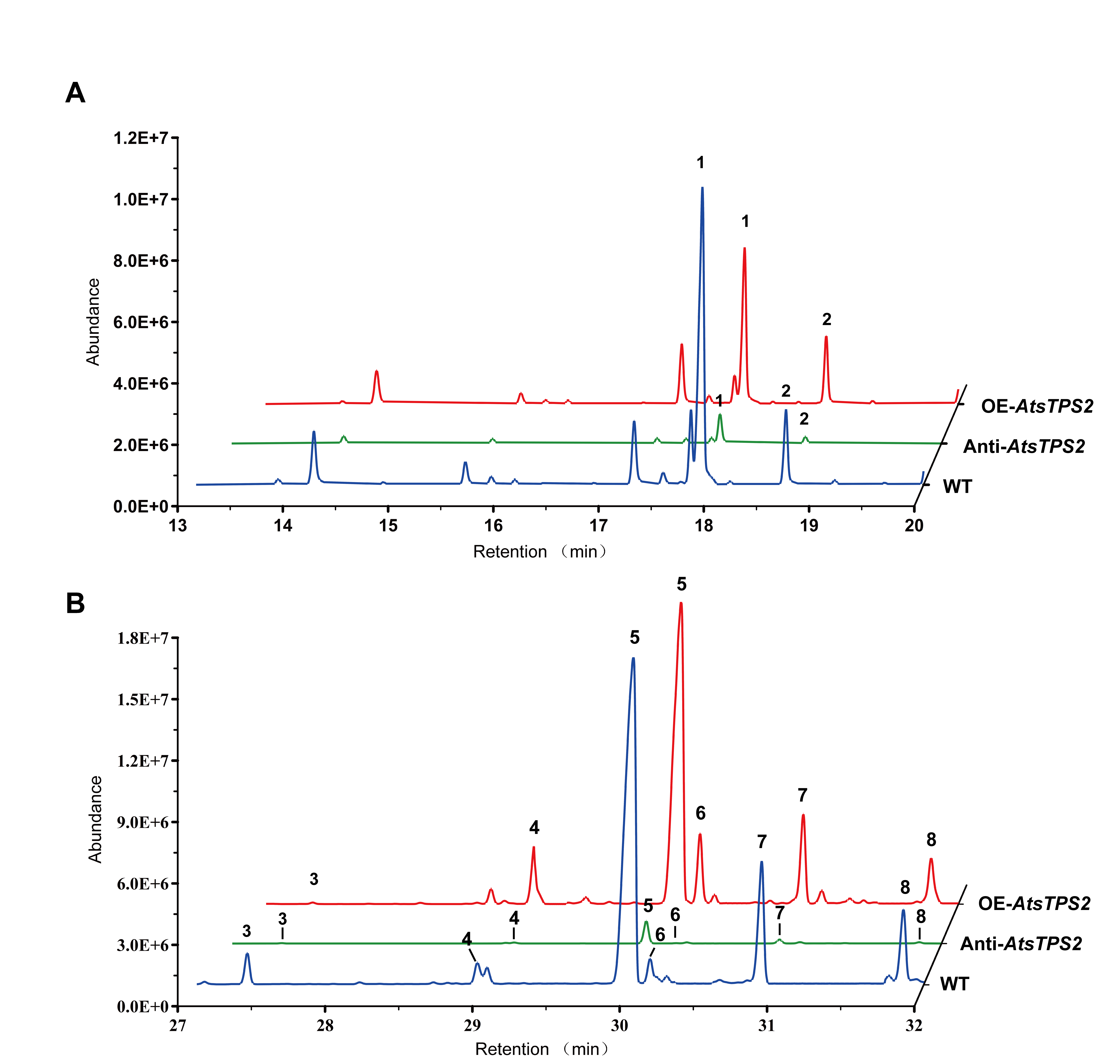


**Supplementary Figure 7.** HS-SPME-GC-MS peak profiles of volatile terpenoids in WT, Anti-*AtsTPS2*, and OE-*AtsTPS2* lines. WT is shown in blue, Anti-*AtsTPS2* in green, and *OE-AtsTPS2* in red. (A) Representative HS-SPME-GC-MS peak profiles of monoterpenes in the three lines. Peaks labeled in panel A are: 1, eucalyptol; 2, *γ*-terpinene. Other volatile signals in this retention time range are not labeled. (B) Representative HS-SPME-GC-MS peak profiles of sesquiterpenes in the three lines. Peaks labeled in panel B are: 3, germacrene B (detected as *γ*-elemene); 4, germacrene A (detected as *β*-elemene); 5, *β*-caryophyllene; 6, (−)-isolongifolol; 7, juniperene; and 8, *α*-caryophyllene.


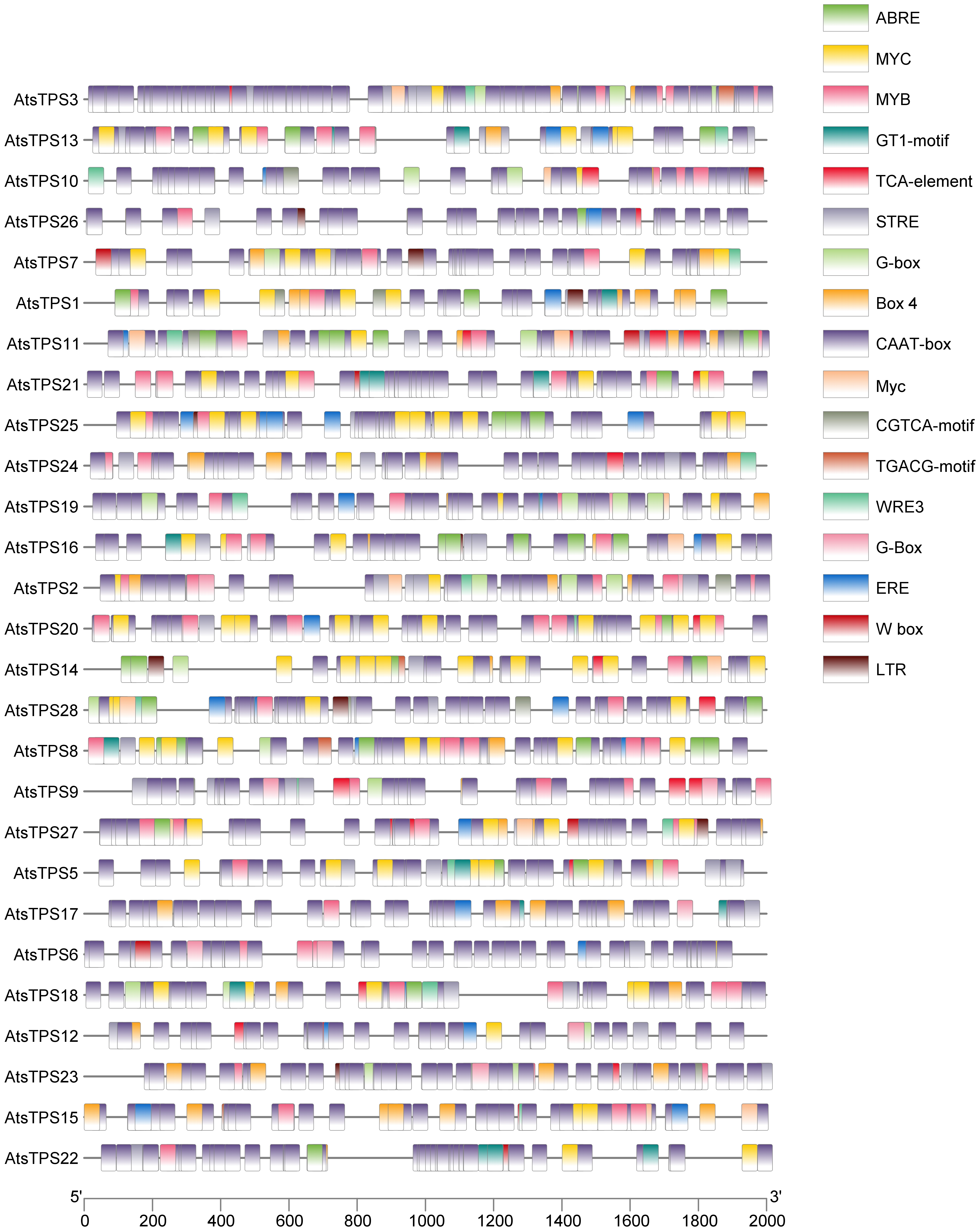


**Supplementary Figure 8.** Cis-acting elements and functions of the *AtsTPSs* promoter.


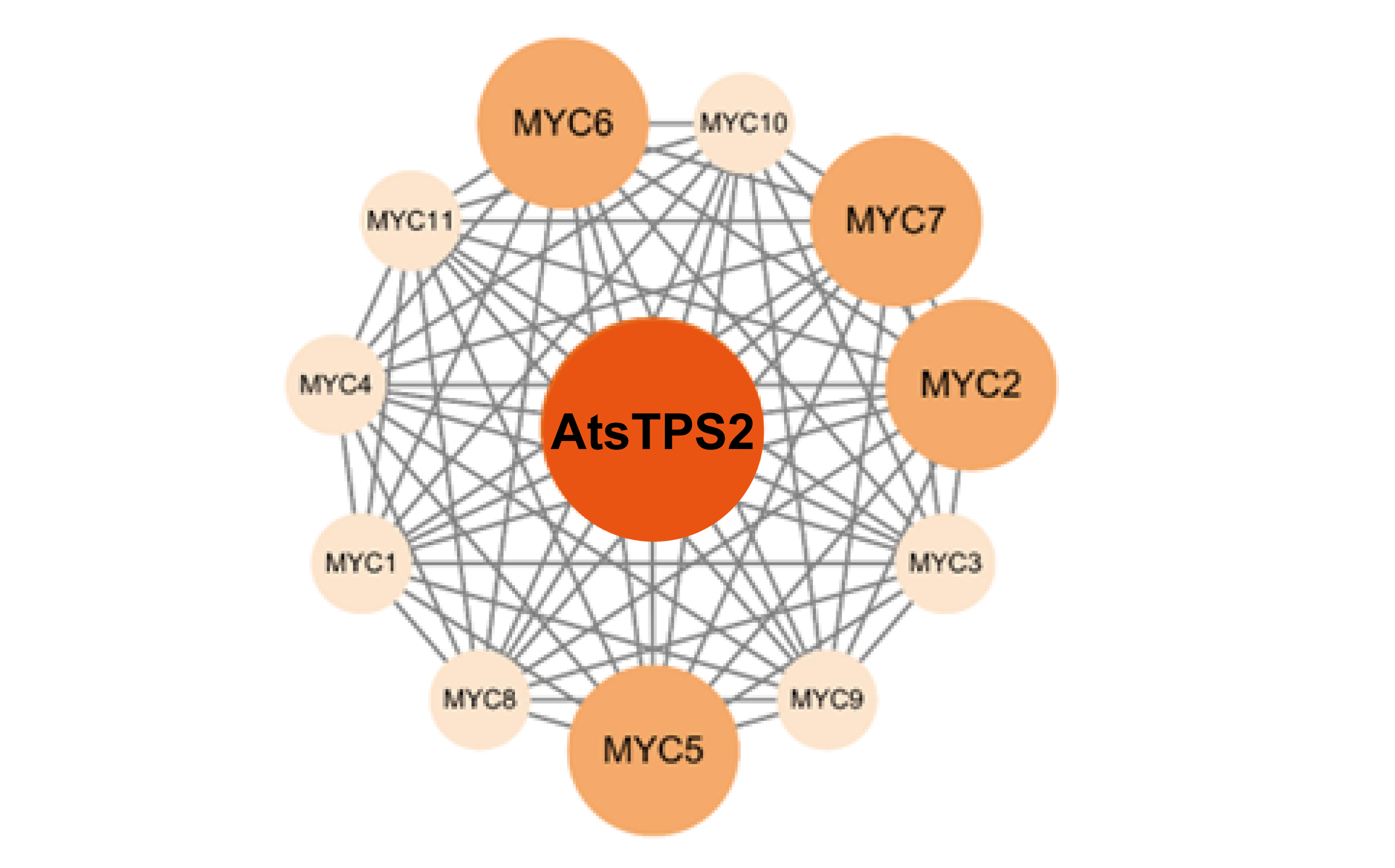


**Supplementary Figure 9.** Co-expression network of *AtsTPS2* gene and AtsMYC transcription factor in *A. tatarinowii.*


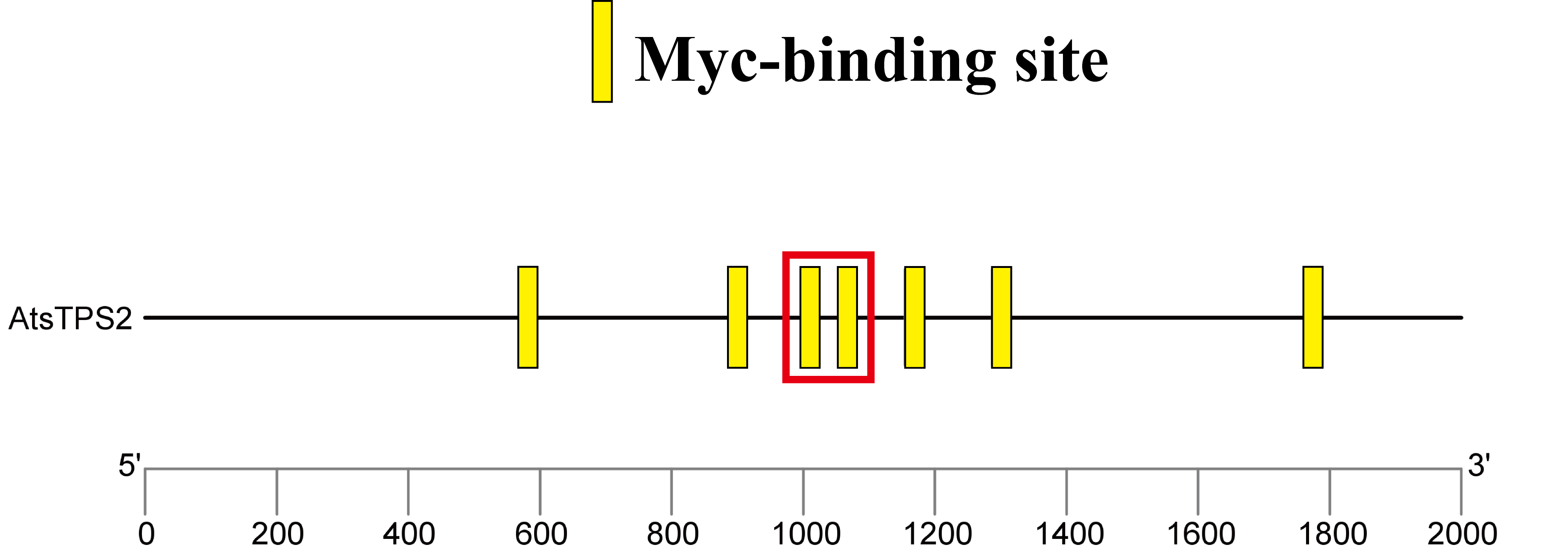


**Supplementary Figure 10.** 120 bp fragment located from positions -1100 to -980 relative to the transcription start site, TSS. Yellow squares represent MYC-binding site, and the red boxes contain the selected fragment.

*
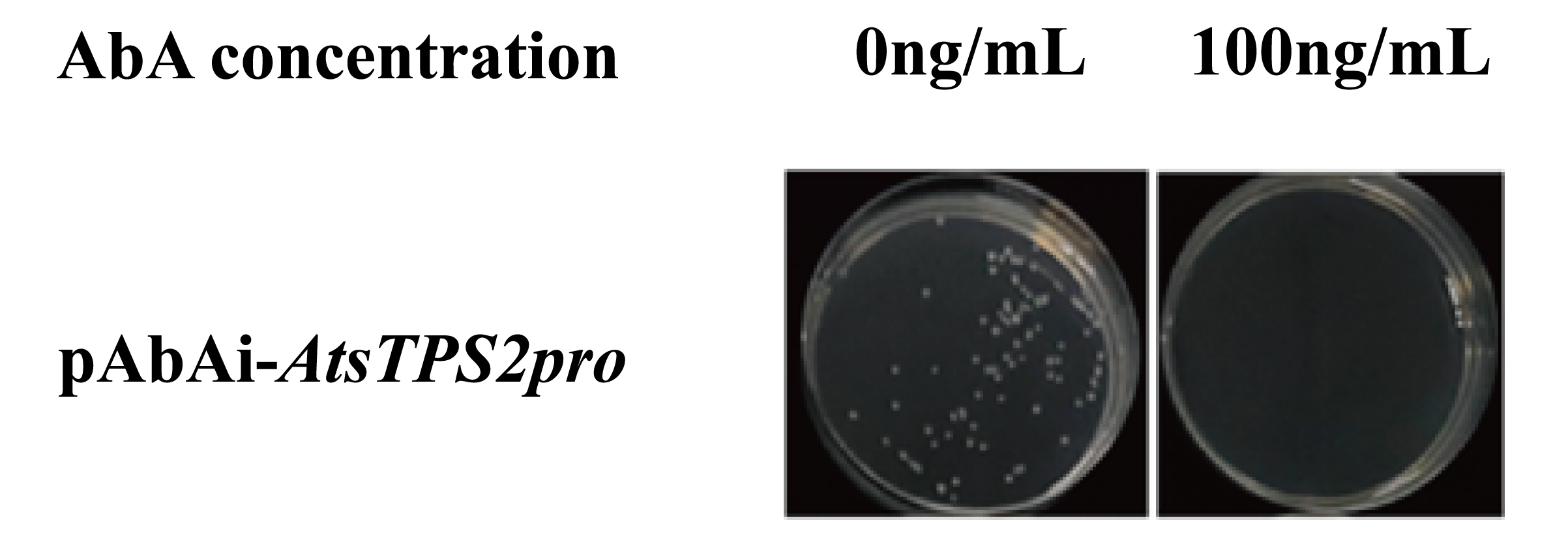
*

**Supplementary Figure 11.** *AtsTPS2* promoter schematic diagram of segmental self-activation.

# Supplementary Tables

**Supplementary Table 1.** Detailed quantitative data of each volatile terpenoid in all samples.

| **Terpenoid** | relative content in leaves  (μg·kg⁻¹ FW·h⁻¹) | | | relative content in rhizomes  (μg·kg⁻¹ FW·h⁻¹) | | |
| --- | --- | --- | --- | --- | --- | --- |
|  | 1a-L | 2a-L | 3a-L | 1a-R | 2a-R | 3a-R |
| Eucalyptol | 140.82 | 65.97 | 24.10 | 86.64 | 18.22 | 21.80 |
| *γ*-Terpinene | 25.78 | 17.43 | - | - | - | - |
| germacrene A | 19.01 | 23.21 | - | 24.65 | - | - |
| *β*-Caryophyllene | 383.08 | 542.88 | 188.14 | - | 613.79 | - |
| (-)-Isolongifolol | 109.78 | 37.49 | 10.83 | 57.39 | 63.61 | 67.88 |
| Juniperene | 30.46 | 152.34 | - | 62.49 | 264.87 | - |
| *α*-Caryophyllene | - | 96.53 | - | - | - | - |
| germacrene B | - | 26.44 | 17.13 | - | - | - |
| 3-Carene | 18.73 | - | 5.15 | 35.35 | 11.07 | 12.36 |
| Camphene | - | - | - | 41.79 | - | 15.49 |
| Sabinene | - | - | - | 32.38 | - | - |
| Cubebol | - | - | - | 233.178 | - | - |
| α-Quercenol | - | - | - | 116.88 | - | - |
| γ-Cedrene | - | - | - | 86.44 | - | - |
| (-)-α-Calarene | - | - | - | 303.16 | 623.68 | 149.16 |
| (+)-γ-Calarene | - | - | - | 771.96 | - | - |
| α-Pinene | - | - | - | - | 322.76 | - |
| β-Juniperene | - | - | - | - | 328.37 | - |
| Calamenene | - | - | - | - | - | 154.73 |

**Supplementary Table 2.** Information of *AtsTPSs* gene family members of *A. tatarinowii.*

| Sequence ID | Number of Amino Acid | Molecular  Weight | Theoretical pI | Instability Index | Aliphatic Index | Grand Average of Hydropathicity |
| --- | --- | --- | --- | --- | --- | --- |
| AtsTPS9 | 594 | 68674.27 | 5.19 | 39.09 | 88.28 | -0.323 |
| AtsTPS10 | 513 | 59766.61 | 5.43 | 43 | 91.97 | -0.225 |
| AtsTPS11 | 610 | 70494.43 | 5.18 | 42.26 | 92.05 | -0.269 |
| AtsTPS20 | 713 | 80776.11 | 5.23 | 52 | 91.89 | -0.201 |
| AtsTPS12 | 543 | 62999.44 | 5.55 | 49.91 | 92.69 | -0.245 |
| AtsTPS23 | 533 | 61819.37 | 7.01 | 43.98 | 84.56 | -0.349 |
| AtsTPS24 | 679 | 77821.17 | 6.25 | 42.34 | 85.73 | -0.316 |
| AtsTPS25 | 586 | 67759.12 | 6.06 | 45.8 | 84.06 | -0.378 |
| AtsTPS13 | 596 | 68956.22 | 5.17 | 48.15 | 85.89 | -0.384 |
| AtsTPS26 | 560 | 64132.84 | 5.83 | 42.35 | 85.7 | -0.258 |
| AtsTPS1 | 371 | 43343.24 | 5.33 | 36.45 | 93.34 | -0.242 |
| AtsTPS2 | 557 | 64327.75 | 5.19 | 45.45 | 98.19 | -0.347 |
| AtsTPS3 | 429 | 50162.91 | 6.07 | 43.78 | 99.58 | -0.321 |
| AtsTPS4 | 470 | 54640.52 | 5.46 | 44 | 93.79 | -0.386 |
| AtsTPS5 | 426 | 49631.17 | 5.16 | 44.99 | 97.75 | -0.275 |
| AtsTPS6 | 585 | 68321.06 | 5.31 | 44.72 | 89.49 | -0.339 |
| AtsTPS7 | 556 | 65143.75 | 5.51 | 44.78 | 90.14 | -0.341 |
| AtsTPS14 | 546 | 62336.1 | 5.94 | 47.93 | 91.83 | -0.162 |
| AtsTPS28 | 490 | 57172.98 | 5.66 | 54.45 | 85.41 | -0.373 |
| AtsTPS27 | 556 | 64308.34 | 6.09 | 55.61 | 86.83 | -0.338 |
| AtsTPS16 | 705 | 82627.97 | 5.25 | 41.25 | 87.84 | -0.354 |
| AtsTPS17 | 768 | 89350.57 | 5.17 | 45.81 | 87.25 | -0.342 |
| AtsTPS15 | 614 | 71149.47 | 5.9 | 43.82 | 91.92 | -0.252 |
| AtsTPS21 | 672 | 76632.44 | 5.49 | 51.43 | 88.35 | -0.267 |
| AtsTPS22 | 759 | 86387.89 | 6.26 | 47.89 | 86.82 | -0.292 |
| AtsTPS18 | 709 | 82266.11 | 5.65 | 40.33 | 89.56 | -0.221 |
| AtsTPS19 | 915 | 105613.5 | 5.83 | 38.75 | 87.81 | -0.319 |
| AtsTPS8 | 545 | 62490.22 | 5.59 | 40.89 | 90.06 | -0.224 |

**Supplementary Table 3.** Terpene Synthase (TPS) Gene IDs and Names of *Acorus tatarinowii* and *Arabidopsis thaliana*

| ID | **Gene Name** | **ID** | **Gene Name** |
| --- | --- | --- | --- |
| EVM0004650 | AtsTPS1 | AtTPS1 | At4g15870 |
| EVM0010886 | AtsTPS2 | AtTPS2 | At4g16730 |
| EVM0001068 | AtsTPS3 | AtTPS3 | At4g16740 |
| EVM0019282 | AtsTPS4 | AtTPS4 | At1g61120 |
| EVM0018588 | AtsTPS5 | AtTPS5 | At2g23230 |
| EVM0019908 | AtsTPS6 | AtTPS6 | At1g70080 |
| EVM0004506 | AtsTPS7 | AtTPS7 | At4g20200 |
| EVM0017523 | AtsTPS8 | AtTPS8 | At4g20210 |
| EVM0017542 | AtsTPS9 | AtTPS9 | At4g20230 |
| EVM0004084 | AtsTPS10 | AtTPS10 | At2g24210 |
| EVM0006012 | AtsTPS11 | AtTPS11 | At5g44630 |
| EVM0020337 | AtsTPS12 | AtTPS12 | At4g13280 |
| EVM0001769 | AtsTPS13 | AtTPS13 | At4g13300 |
| EVM0014378 | AtsTPS14 | AtTPS14 | At1g61680 |
| EVM0021069 | AtsTPS15 | AtTPS15 | At3g29190 |
| EVM0008005 | AtsTPS16 | AtTPS16 | At3g29110 |
| EVM0018643 | AtsTPS17 | AtTPS17 | At3g14490 |
| EVM0019947 | AtsTPS18 | AtTPS18 | At3g14520 |
| EVM0007237 | AtsTPS19 | AtTPS19 | At3g14540 |
| EVM0012965 | AtsTPS20 | AtTPS20 | At5g48110 |
| EVM0006073 | AtsTPS21 | AtTPS21 | At5g23960 |
| EVM0022311 | AtsTPS22 | AtTPS22 | At1g33750 |
| EVM0020961 | AtsTPS23 | AtTPS23 | At3g25830 |
| EVM0007065 | AtsTPS24 | AtTPS24 | At3g25810 |
| EVM0006136 | AtsTPS25 | AtTPS25 | At3g29410 |
| EVM0004500 | AtsTPS26 | AtTPS26 | At1g66020 |
| EVM0018027 | AtsTPS27 | AtTPS27 | At3g25820 |
| EVM0015601 | AtsTPS28 | AtTPS28 | At1g48800 |
|  |  | AtTPS29 | At1g31950 |
|  |  | AtTPS30 | At3g32030 |
